# Magnetite drives microbial community restructuring and stimulates aceticlastic methanogenesis of type II *Methanosarcina *in mangrove sediments

**DOI:** 10.1186/s40168-025-02157-z

**Published:** 2025-07-26

**Authors:** Jinjie Zhou, Cui-Jing Zhang, Dayu Zou, Chengxiang Gu, Meng Li

**Affiliations:** Archaeal Biology Centre, Synthetic Biology Research Center, Shenzhen Key Laboratory of Marine Microbiome Engineering, Key Laboratory of Marine Microbiome Engineering of Guangdong Higher Education Institutes, Institute for Advanced Study, Shenzhen University, Shenzhen, 518060 China

**Keywords:** Methane, Magnetite, *Methanosarcina*, Aceticlastic methanogenesis, Hydrogenotrophic methanogenesis, Mangrove sediments, Metagenome, Metatranscriptome

## Abstract

**Background:**

Mangrove wetlands are critical hotspots of methane emissions, yet the role of naturally occurring minerals in shaping their microbial communities and methanogenic processes is poorly understood. Magnetite, a common iron mineral in soils and sediments, has been reported to enhance aceticlastic methanogenesis and facilitate syntrophic methanogenesis. In this study, we integrated multi-omic profiling with cultivation-based approaches to investigate the impact of magnetite on methanogenesis of microbial consortia derived from mangrove sediments, using lactate as a substrate.

**Results:**

Across five serial transfers, mangrove microbial consortia converted lactate to propionate and acetate, which were subsequently degraded into methane. Magnetite addition significantly stimulated methane production, leading to notable changes in community structure, particularly for aceticlastic methanogens, with *Methanosarcina* predominating in the magnetite-amended cultures and *Methanothrix* in controls. Four *Methanosarcina* strains T3, T4, T13, and MeOH were subsequently isolated from magnetite-amended cultures. Combined analyses of metagenome-assembled genomes and the genomes of these isolates revealed that the enriched *Methanosarcina* in magnetite-amended cultures belonged to type II deficient in hydrogenotrophic methanogenesis pathway. Metatranscriptomic analyses suggested that magnetite addition stimulated aceticlastic methanogenesis of type II *Methanosarcina* and hydrogenotrophic methanogenesis of *Methanomicrobiales* in the consortia. Furthermore, pure culture experiments confirmed that magnetite stimulated aceticlastic methanogenesis by *Methanosarcina* sp. T3, although its gene expression patterns differed from those observed in the microbial consortia. Additionally, *Methanofastidiosales*, an uncultured archaeal lineage possessing H_2_-dependent methylotrophic methanogenesis, was detected in all transfers.

**Conclusions:**

Our findings demonstrate that magnetite alters methanogenic consortia in mangrove sediments, selectively stimulating aceticlastic methanogenesis of type II *Methanosarcina* and modulating hydrogenotrophic activity in *Methanomicrobiales*. By integrating multi-omics analyses with pure culture validation, we demonstrate, for the first time, that magnetite directly enhances the aceticlastic methanogenesis of type II non-hydrogenotrophic *Methanosarcina*. This study provides new insights into the influence of magnetite on complex microbial consortia, offers a deeper understanding of the physiology of type II non-hydrogenotrophic *Methanosarcina*, and advances knowledge of mineral-mediated regulation of methanogenic networks in anoxic environments.

Video Abstract

**Supplementary Information:**

The online version contains supplementary material available at 10.1186/s40168-025-02157-z.

## Introduction

Methane is the second most significant greenhouse gas after CO_2_ contributing to global warming and climate change [1, 2]. Since 2007, atmospheric methane concentrations have risen steadily, primarily due to biogenic activities [3, 4]. Aquatic ecosystems, such as wetlands, lakes, rice paddies, and coastal seas are significant sources of methane, accounting for half of total global methane emissions [5]. Among this, mangrove wetlands located in tropical and subtropical estuaries are significant natural sources of methane. Despite covering only 0.5% of the coastal area, mangroves contribute 10–15% of total carbon storage, making them critical “blue carbon” for carbon capture and storage [6, 7]. However, methane emissions from mangrove sediments partially offsets their carbon sequestration benefits [8]. Understanding the biogenic methanogenesis process in mangrove sediments is therefore crucial for evaluating their climate impact and role in global carbon cycle.

Methanogenic archaea are the primary biological producers of methane. They metabolize a narrow range of substrates, primarily acetate (aceticlastic), H_2_/CO_2_ (hydrogenotrophic), and methyl compounds (methylotrophic) [9, 10], with novel substrates continually being discovered [11, 12]. Among these pathways, aceticlastic methanogenesis is considered the dominant pathway, contributing to two-thirds of global biogenic methane emissions, followed by hydrogenotrophic methanogenesis, which accounts for one-third [13]. Fermentative and syntrophic bacteria sequentially break down complex organic matter into short-chain fatty acids (e.g., propionate, butyrate), then to acetate and H₂/CO₂. These products are rapidly consumed by methanogens, and their removal is essential to maintain overall degradation process thermodynamically favorable for bacteria [14].

*Methanothrix* (formerly *Methanosaeta*) and *Methanosarcina* are the primary aceticlastic methanogens, but the two taxa exhibit significant physiological and ecological differences: *Methanothrix* within the family *Methanotrichaceae* is an aceticlastic specialist with high acetate affinity (5–20 μM), making it a strong competitor in low acetate environments. In contrast, *Methanosarcina* is metabolically versatile with a higher growth rate but requires a higher acetate threshold (~ 1 mM) [15, 16]. Notably, *Methanosarcina* can be further divided into two types with distinct energy conservation strategies [17]: Type I hydrogenotrophic *Methanosarcina* (e.g., *Methanosarcina barkeri*), utilize H_2_ cycling by energy-converting hydrogenases for energy conservation during growth on acetate [18], and are predominant in organic-rich environments like anaerobic digester. While type II *Methanosarcina* (e.g., *Methanosarcina acetivorans*) are typically non-hydrogenotrophic, using Rnf (Rhodobacter nitrogen fixation) complex to generate a sodium ion gradient for energy conservation during aceticlastic methanogenesis [19], thriving in organic-poor anaerobic soils and sediments. These physiological differences shape their distribution and competitive success in different environmental conditions.

Magnetite (Fe_3_O_4_), a naturally abundant mixed-valence iron mineral, is widely found in soils and sediments [20, 21]. It can be synthesized both abiotically or biotically, acting as electron sinks or electron sources in various microbial processes [22, 23]. Notably, magnetite has been shown to enhance methanogenesis especially for aceticlastic methanogens. On the one hand, magnetite stimulates aceticlastic methanogenesis of type I *Methanosarcina* including *M*. *barkeri* and *Methanosarcina mazei* [24, 25], and *Methanothrix thermoacetophila* [26], possibly serving as an electron shuttle to facilitate intracellular/extracellular electron transfer. On the other hand, magnetite stimulates syntrophy between bacteria and methanogens, potentially replacing membrane-bound cytochrome *c* to promote direct interspecies electron transfer (DIET) from bacteria to methanogens for CO_2_ reduction to methane [27]. Magnetite supplementation has been frequently used to enhance methanogenesis in anaerobic digesters and microbial electrochemical systems [28–31]. However, the ecological relevance and community-level impacts of magnetite supplementation in natural anoxic environments has not been well defined.

In mangrove sediments, pulses of organic carbon from tidal inputs or root exudates, along with iron-rich depositional zones, can elevate the availability of both electron donors and redox-active minerals such as magnetite [32, 33]. These conditions may significantly influence microbial community structure and methanogenic pathways. In this study, we investigated the effect of magnetite supplementation on methanogenic activity and community structure in microbial consortia from mangrove sediments, using lactate as substrate to simulate fermentative conditions. By combining metagenomic and metatranscriptomic analyses with cultivation-based approaches, we demonstrated that magnetite not only enhanced methanogenesis, but also shifted community composition, favoring type II *Methanosarcina* and modulating the expression of hydrogenotrophic pathways in *Methanomicrobiales*. These findings underscore the ecological importance of type II *Methanosarcina* in magnetite-enriched anoxic environments, and provide new insights into how naturally occurring minerals influence methane emissions from mangrove ecosystems.

## Methods

### Cultivation

The mangrove sediment samples (0–2 cm depth) were collected from MG1 site in Shenzhen Futian Mangrove Reserve (22.31°N 114.00°E) in May 2021. After collection, the sediment samples were immediately stored into a sealed box with an AnaeroPack sachet (Mitsubishi), taken to the laboratory, and stored at 4 °C for 2 months.

The basal medium used in the current study was a modification of DSMZ 141c medium, in which MgSO_4_·7H_2_O, yeast extract, and trypticase peptone were excluded from the medium to slow the growth of sulfate-reducing and fermentative bacteria [34]. l-lactate (20 mM) was used as the substrate instead of methanol/trimethylamine/acetate. When noted, nanoscale magnetite (10 mM), prepared as previously described [27], was added anaerobically to the medium before autoclaving. Approximately 0.5 g of sediment was placed in 125-mL serum bottles (Wheaton) with 50 mL of anaerobic basal medium (*n* = 3 in each group) in an anaerobic glove box (Shell Lab), followed by replacement of the headspace gas with N_2_/CO_2_ mixture (80/20, v/v) for 30 min. The culture was incubated at 30 °C. All cultures were subjected to five successive transfers (referred to as generations), each at a 10% inoculum volume, using a syringe equipped with a 23G (0.6 × 25 mm) needle pre-flushed with N₂. Samples were collected and analyzed at the 1st, 3rd and 5th generations. For multi-omics analyses, cultures were sampled at two stages: the initial stage (E) when lactate was completely consumed, and the mid-logarithmic stage (L) when methane production reached approximately 18 mM. Sample labels are defined as follows: CE and CL represent stage E and L for the control group, respectively, while CME and CML represent the corresponding stages for the magnetite-amended group.

### Analytics

Methane concentration was determined by gas chromatography (Shimadzu, GC-2014) equipped with an SH-Rtx-5 column and a flame ionization detector (FID). The injector, column, and detector temperatures were set at 150, 80, and 200 °C, respectively. Hydrogen concentration was determined by gas chromatography (Techcomp, GC7900) equipped with a TDX-01 column and a thermal conductivity detector (TCD). The column and detector temperatures were set at 60 °C and 150 °C, respectively, and the bridge current was 60 mA. Concentrations of organic acids were determined by high-performance liquid chromatography (HPLC, Agilent 1260 Infinity) equipped with an Aminex HPX-87H ion exclusion column (Bio-Rad), using 5 mM H_2_SO_4_ solution as mobile phase at a flow rate of 0.6 mL/min and a column temperature of 35 °C.

### DNA and RNA extraction

Genomic DNA from mangrove sediment was extracted with a DNeasy PowerSoil kit (Qiagen, Germany). For DNA extraction from enrichments, triplicate cultures (2 mL) were centrifuged at 18,000 g for 5 min at 4 °C. The genomic DNA of the cell pellets was extracted with a MasterPure Complete DNA Purification Kit (Lucigen).

For RNA extraction, triplicate cultures were harvested at stage L (CL, CML) at 4,500 g at 4 °C for 20 min. The cell pellets were immediately frozen with liquid nitrogen and stored at − 80 °C. Total RNA was extracted as previously described [35].

### 16S rRNA gene amplification and analysis

The V4-1 region of the prokaryotic 16S rRNA gene sequences was amplified using primers 515F (5′-GTGCCAGCMGCCGCGGTAA-3′) and 806R (5′-GGACTACHVGGGTWTCTAAT-3′). The sequencing libraries were generated using the NEBNext® UltraTM II DNA Library Prep Kit for Illumina® (New England Biolabs, MA, USA), and sequenced on an Illumina Nova6000 platform and 250 bp paired-end reads were generated (Magigene, Guangzhou, China). Data were processed via QIIME2 (ver. 2024.10) [36] using the SILVA 138 99% OTUs full-length sequences database. Taxonomic assignments were refined using GTDB-Tk r220 database, with corresponding name from the SILVA database provided in parentheses at first mention. Alpha diversity was calculated using the core-metrics-phylogenetic method in QIIME2. Principal coordinate analysis (PCoA) based on Bray–Curtis dissimilarity was performed using all feature counts with ChiPlot (https://www.chiplot.online).

### Metagenomic assembly and binning

Metagenomic libraries were generated by ALFA-SEQ DNA Library Prep Kit following manufacturer’s recommendations, and sequenced on an Illumina NovaSeq 6000 platform and 150 bp paired-end reads were generated (Magigene, Guangzhou, China).

Raw reads were trimmed with Sickle (ver. 1.33, -q 25) (https://github.com/najoshi/sickle). Trimmed reads from four samples in the 5th generation (CE, CL, CME, CML) were assembled separately or together with four assemblers, named metaSPAdes (ver. 3.15.0, k21,33,55,77,99,121) [37], IDBA-UD (ver. 1.1.1 –mink 66 –maxk 146 –step 3) [38], IDBA-Hybrid (ver. 1.1.1 –mink 66 –maxk 146 –step 3), and MEGAHIT (ver. 1.1.3 –meta-sensitive, –min-contig-len 1000) [39]. The assembled contigs were then binned by MetaWRAP binning module (ver. 1.3.2 –MetaBat, –MetaBat2, –MaxBin2 –universal, –concoct) [40]. The produced bins were refined by MetaWRAP bin refinement module (ver. 1.3.2) with > 50% completeness and < 10% contamination. All bins were then dereplicated with dRep (ver. 2.2.3) [41], resulting in 112 metagenome assembled genomes (MAGs) with completeness > 50% and contamination < 7.2%. In addition, in order to obtain *Methanosarcina* MAGs, reads that were mapped to genomes of *Methanosarcina* type species were retrieved from trimmed reads by bwa-mem (ver. 0.7.5a-r405) [42], and reassembled with the above reference genome. The assembled contigs were then binned with the MetaWRAP binning module mentioned above. *Methanosarcina* MAGs with completeness higher than 90% were refined by Magpurify (ver. 2.1.2) [43] and Anvi’o 7 [44] to remove contaminating contigs. After dereplication with dRep (ver. 2.2.3), five medium-quality *Methanosarcina* MAGs (completeness > 50%, contamination < 20%) were obtained.

### Isolation of *Methanosarcina*

Isolation of *Methanosarcina* species from enrichments amended with magnetite was carried out using the above basal medium with methanol (100 mM) or trimethylamine (50 mM) instead of lactate as the substrate. Four antibiotics (tetracycline (20 μg/mL), neomycin (200 μg/mL), spectinomycin (20 μg/mL), and streptomycin (200 μg/mL)) were added to the medium from anoxic filter-sterile stocks to inhibit bacterial growth. After three rounds of enrichments, dilution-to-extinction was preformed, and cultures from the highest dilutions showing growth and methane production were streaked on solid medium with four antibiotics and 1.5% Noble agar. After 2 weeks, single colonies were picked and transferred to the liquid medium. Purification of methanogen was confirmed by 16S rRNA gene sequencing using primers Arc21F and 1492R [34]. Possibilities of bacterial contamination were ruled out by microscopy and by PCR amplification with 16S rRNA genes targeting bacteria [34]. As a result, four methylotrophic methanogens, named T3, T4, T13, and MeOH, were successfully isolated from the magnetite-amended enrichment. To determinate whether the *Methanosarcina* isolate performed hydrogenotrophic methanogenesis, strain T3 was grown with H_2_ or formate (40 mM) as the electron donor with CO_2_ as the electron acceptor. H_2_ was provided as an H_2_/CO_2_ mixture (80/20, v/v; 1 overpressure). Growth was monitored by changes in optical density at 600 nm and by methane production. For morphological analysis, cell pellets of strains T3 and MeOH growing under their late-exponential phase were prepared as previously described [26] and observed with a field emission scanning electron microscope (Hitachi Regulus 8100) at 3.0 kV.

### Genome assembly of *Methanosarcina* isolates

The genomic DNA of four *Methanosarcina* was extracted as previously described and sequenced on an Illumina NovaSeq 6000 PE150 platform (Magigene, Guangdong, China). Raw reads were filtered using BBDuk (https://sourceforge.net/projects/bbmap/). The trimmed reads were assembled with SPAdes (ver. 3.13.1) with the –sc and –careful options [45]. Contigs with a length shorter than 500 bp or a coverage lower than 100 × were discarded.

### Genome annotation

The 117 assembled MAGs and genomes of *Methanosarcina* isolates were taxonomically classified with GTDB-Tk (ver. 2.4.0, database r220) [46]. Coding genes were predicted with Prodigal (ver. 2.6.3, -p meta for MAGs) [47], and annotated by EggNOG-mapper (ver. 2.1.9) [48] and KEGG Automatic Annotation Server (KAAS) [49]. The 16S rRNA genes were predicted by barrnap (ver. 0.9) [50].

### Phylogenetic analysis

Phylogenetic analysis of genomes from metagenomic assembly and isolates were based on alignment of 120 bacterial or 53 archaeal single-copy marker genes from GTDB-Tk (ver. 2.4.0, database r220). Amino acid sequences of McrA were extracted by HMMER (ver. 3.3.2) for MCR_alpha (PF02249) and MCR_alpha_N (PF02745) with *E* value of 1E-5 (http://hmmer.org), aligned with MUCSLE (ver. 3.8.31) [51], and trimmed by trimAl (ver. 1.4.1, -gt 0.9) [52]. The phylogenetic trees based on single-copy marker genes or McrA proteins (> 300 amino acids) were constructed by FastTree (ver. 2.1.11) using default parameters [53], and modified with the Interactive Tree of Life (iTOL) [54].

### Metatranscriptomic sequence and analysis

The whole mRNAseq libraries were generated by NEB Next® UltraTM Nondirectional RNA Library Prep Kit for Illumina® (New England Biolabs, MA, USA), and sequenced on the Illumina NovaSeq 6000 PE150 platform (Magigene, Guangzhou, China).

Raw metatranscriptome and transcriptomic data were trimmed with Sickle and Trimmomatic (ver. 0.39) [55], respectively. Ribosomal RNA (rRNA) reads were then removed from the libraries with SortMeRNA (ver. 4.3.4) [56]. The mRNA reads were then mapped against the CDS database obtained from the constructed MAGs and genome of *Methanosarcina* isolate T3 with bwa-mem (ver. 0.7.17-r1188). The gene expression levels were normalized by Transcript per million (TPM) values. Reads were processed for differential expression studies using the R-Package edgeR (ver. 3.40.2) [57]. Genes that were ≥ twofold differentially expressed (|Log_2_FC|> 1) with false discovery rate (FDR) of ≤ 0.05 are summarized in Supplementary Table S4 and S6.

### Relative abundance of genomes in metagenomic and metatranscriptomic libraries

The relative abundances of 117 MAGs and 1 *Methanosarcina* sp. T3 genome in the metagenomic and metatranscriptomic datasets were calculated using CoverM (ver. 0.6.1, -p minimap2-sr -m relative_abundance –min-read-aligned-percent 0.75 –min-read-percent-identity 0.95 –min-covered-fraction 0) [58]. As a result, 52.3–70.1% of the metagenomic reads and 39.7–56.5% of the metatranscriptomic reads could be mapped to the 118 genomes.

### Statistical analysis

Differences in methane production and bacterial relative abundance in metatranscriptomic libraries between the magnetite-amended and control groups were determined by two-tailed paired *t* tests. For alpha diversity, statistical significance among groups was determined by QIIME2 using Kruskal–Wallis (pairwise) method via alpha-group-significance command [36]. Differences were considered statistically significant at *P* < 0.05.

## Results

### Magnetite stimulated methanogenesis of mangrove microbial consortia

To evaluate the effects of magnetite on methanogenic process of complex microbial communities from mangrove sediments, lactate was selected as the sole substrate. Although rare in the environment, lactate is one of the major intermediates of fermentative bacteria from numerous carbohydrates, and can be easily converted into acetate, propionate, CO_2_, and hydrogen by numerous anaerobic bacteria, and ultimately into methane by methanogens [59].

**Fig. 1:**
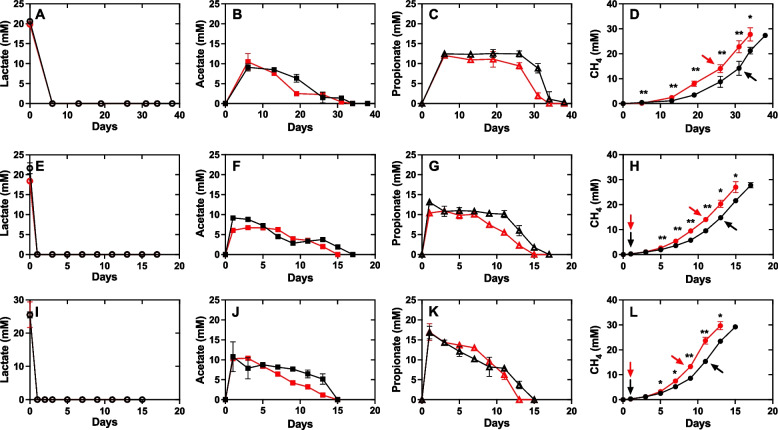
Effect of magnetite on methane production of mangrove microbial consortia using lactate as substrate at 1st (**A**–**D**), 3rd (**E**–**H**), and 5th (**I**–**L**) generation in the presence (red lines) or absence (black lines) of magnetite. Arrows indicated the sampling timepoints for 16S rRNA gene amplicon sequencing (1st, 3rd, and 5th generation), and for metagenomic and metatranscriptomic sequencing (5th generation). Data represent mean values and standard deviations from three independent cultures. Difference in methane production between the two groups were tested by two-tailed paired *t* tests, indicated by * when *p* < 0.05, or **p < 0.01

In both the magnetite-amended and control groups, the lag phase of methane production in mangrove microbial consortia decreased from 13 to 3 days after four successive transfers, indicating rapid enrichment of the functional methanogenic consortia. Methane yields of 1.15–1.47 mol/mol lactate for 3rd and 5th transfer were consistent with the overall stoichiometry (2 C_3_H_6_O_3_ → 3 CH_4_ + 3 CO_2_), considering that some carbon was used for biomass production (Fig. 1).

At the start of cultivation when lactate was completely consumed, acetate and propionate were accumulated to maximum values of ~ 10 mM and 13–16 mM, respectively, and subsequently declined along with methane accumulation (Fig. 1). Trace amount of hydrogen (< 1 mM) was detected in the headspace of the 5th-generation cultures (Fig. S1). Formate and butyrate concentrations were below the HPLC detection limit (< 10 μM). These results are consistent with the fact that the conversion of lactate to acetate and propionate is thermodynamically favorable for various bacteria [60, 61]. Thus, magnetite did not affect the lactate metabolic profiles of mangrove microbial consortia, as similar lactate consumption, methane yield, and intermediate formation were observed between the two groups.

Nevertheless, magnetite addition significantly stimulated the methanogenesis of mangrove microbial consortia, resulting in 13.3 ± 8.6%, 10.1 ± 4.9%, and 17.1 ± 5.6% higher methane production rates in the 1st, 3rd, and 5th generations, respectively (Fig. 1). However, unlike a previous study that used acetate as the substrate [24], magnetite addition did not significantly shorten the lag time of methane production (12 days for the 1st transfer, 3 days for the 3rd and 5th transfers in both groups).

### Magnetite affected community structure of mangrove microbial consortia

To evaluate the impact of magnetite on community structure, 16S rRNA gene amplicons were sequenced from the 1st, 3rd, and 5th generations of the magnetite-amended (CM) and control (C) groups. For the 1st generation, samples were collected at the stage L (CL and CML). For the 3rd and 5th generations, samples were collected at both stage E (CE, CME) and stage L (CL, CML). Alpha diversity, as measured by Shannon and Pielous’s indices, decreased with increasing generation, indicating that the serial transfers led to a simplification of community structure and an enrichment of functional microorganisms (Fig. S2). Principal coordinate analysis (PCoA) revealed clear separation in the beta diversity of microbial communities between groups C and CM in each generation (Fig. S3).

**Fig. 2:**
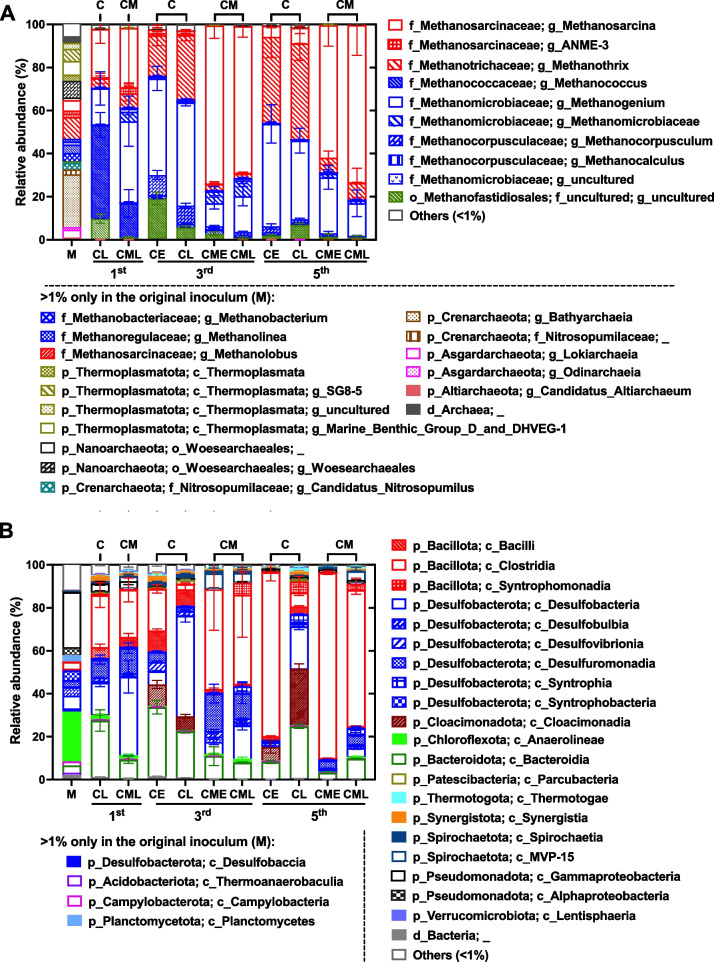
Effects of magnetite on relative abundances of archaea (**A**) and bacteria (**B**) at 1st, 3rd, and 5th generation. Error bars represent standard deviations from three independent cultures. *C* control group without magnetite addition, *CM* magnetite-amended group. M: original inoculum. Only taxa with a relative abundance greater than 1% in at least one group are shown in the figure

In all generations, the proportion of archaea is much lower than that of bacteria, with a higher proportion of archaea observed in stage L (15.4–25.8% of total prokaryotes) compared to stage E (1.0–8.6%) in the 3rd and 5th generations (Fig. S4). The results were consistent with the fact that methane was produced by archaeal methanogens, which were predominantly accumulated in stage L (Fig. 1 H and L). Ten archaeal genera were identified with relative abundances greater than 1% of total archaea, including six hydrogenotrophic genera within the families *Methanomicrobiaceae* (3 genera), *Methanocorpusulaceae* (2 genera), and *Methanococcaceae* (1 genus), three aceticlastic genera within the families *Methanosarcinaceae* (*Methanosarcina*, ANME-3) and *Methanotrichaceae* (formerly *Methanosaetaceae*, *Methanothrix*), and one genus within the H_2_-dependent methylotrophic *Methanofastidiosales* order (Fig. 2).

Magnetite addition resulted in significant differences in archaeal community structure, particularly for aceticlastic methanogen. *Methanosarcina* was the predominant aceticlastic genus in magnetite-amended groups, accounting for 70.7% and 67.3% of the archaeal sequences in the 3rd and 5th generations, respectively, while it occupied only 1.5% and 6.1% in the control group. Conversely, *Methanothrix* constituted 24.2% and 42.3% of the archaeal community in the control group in the 3rd and 5th generations, respectively, but only 2.6% and 7.3% in the magnetite-amended groups (Fig. 2, numbers obtained from average values of stage E and L). Among hydrogenotrophic methanogens, *Methanogenium* was a significant one in all transfers in both groups, while *Methanococcus* was abundant only in the 1st generation, accounting for 43.4% and 15.9% of total archaea at stage L in the control and magnetite-amended groups, respectively. However, these proportions decreased sharply during serial transfers, dropping to less than 2% in the 3rd generation, and disappearing by the 5th generation in both groups.

In contrast, bacterial diversity was greater than archaeal diversity. A total of 11 phyla were detected with relative abundances greater than 1% of total bacteria (Fig. 2B). This is expected, as lactate is an ideal carbon source and electron donor for many fermenters and acetogens. The bacterial community structure was similar at stages E and L for the magnetite-amended group; however, the bacterial structure in the control group differed significantly between the two sampling stages. For example, *Clostridia* was a significant class at stage E, but *Desulfobacteria* predominated at stage L in both the 3rd and 5th generations. *Bacillota* (formerly *Firmicutes*), *Desulfobacterota*, and *Bacteroidota* were enriched in both the control and magnetite-amended groups, whereas *Cloacimonadia* were only detected in the control group, accounting for 26.2% of the bacterial community at stage L in the 5th generation.

An explicit role of magnetite in promoting methanogenesis is its function as an alternative to the outer-membrane *c*-type cytochrome of bacteria, facilitating DIET with methanogens for reduction of CO_2_ to methane [27]. *Geobacter*, a representative genus within the family *Geobacteraceae* and the class *Desulfuromonadia*, has been reported to frequently enriched in magnetite-amended methanogenic communities from various natural and artificial environments [29, 62]. However, in our study, *Geobacteraceae* occupied only a small proportion (< 2% of the total bacteria) in both the magnetite-amended and control groups, with their relative abundance gradually decreasing through successive transfers (Fig. S5). Although *Geobacteraceae* occupied a higher proportion in magnetite-amended group at 3rd and 5 th generations, their contribution to methane production remained unclear in the present study.

### Metagenome assembly and binning

**Fig. 3:**
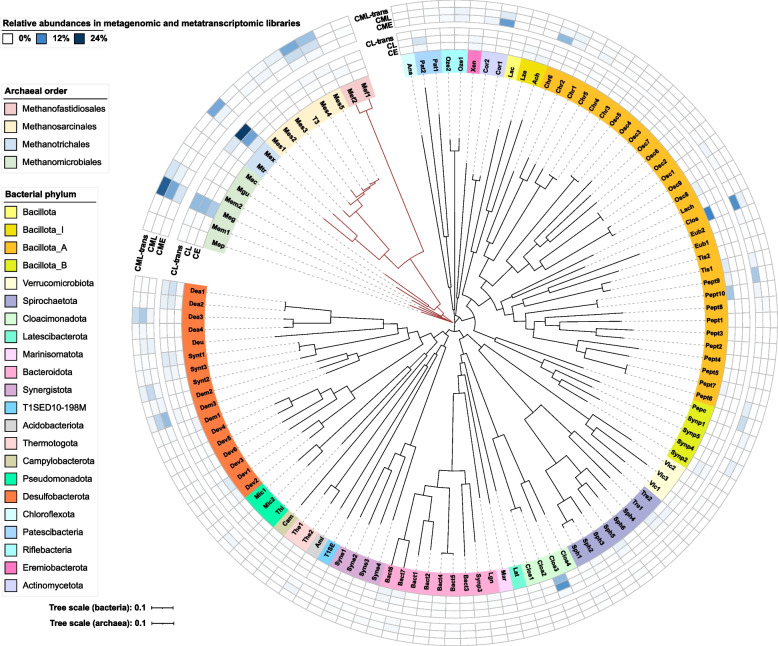
Phylogenetic trees of bacterial and archaeal genomes recovered from metagenome and *Methanosarcina* isolate T3 based on 120 bacterial and 53 archaeal single-copy marker proteins, and the relative abundances of each genome in metagenomic (CE, CL, CME, CML) and metatranscriptomic (CL-trans, CML-trans) libraries in the absence and presence of magnetite in the 5th generation. CE and CL, stage E and L for the control group; CME and CML, stage E and L for the magnetite-amended group. Archaeal tree is presented in brown

To better understand the enriched microbial communities and their methanogenic processes, four DNA samples extracted from the magnetite-amended cultures (CM) and the control cultures (C) at two stages in the 5th generation were sequenced for metagenomic analyses. After using various binning methods and dereplication (see “ Methods” section), a total of 117 MAGs were recovered, including 102 bacterial genomes and 15 archaeal genomes according to the GTDB-Tk database (Fig. 3, Fig. S6, Fig. S7, and Table S1).

For bacteria, 102 high-quality MAGs were reconstructed with > 50% completeness and < 7.2% contamination, which were grouped into 22 phyla (Fig. 3). *Bacillota* (*Bacillota*, *Bacillota*_I, *Bacillota*_A, and *Bacillota*_B) and *Desulfobacterota* were the predominant phyla, accounting for 39 and 17 of the total bacterial MAGs, respectively, consistent with the 16S rRNA gene amplicon analysis (Fig. 2). In contrast, 10 high-quality archaeal MAGs with > 84% completeness and < 2.7% contamination were obtained, assigned to the orders *Methanomicrobiales* (6 MAGs), *Methanotrichales* (2 MAGs), and *Methanofastidiosales* (2 MAGs) (Table S1). However, we were unable to reconstruct high-quality *Methanosarcina* MAGs despite their high abundance in magnetite-amended microbial consortia. Instead, 5 medium-quality *Methanosarcina* MAGs with completeness of 51.86–80.94% and contamination of 12.09–17.65% were obtained by manual refinement (Table S1).

### Isolation of *Methanosarcina* from magnetite-amended microbial enrichments

The 16S rRNA gene amplicon sequencing indicated that the addition of magnetite led to the rapid enrichment of *Methanosarcina* (Fig. 2A). We aimed to isolate *Methanosarcina* from magnetite-amended microbial consortia, to compensate for the unsatisfactory quality of *Methanosarcina* MAGs from metagenome assembly, and to verify the actual effects of magnetite on the growth and methanogenesis of *Methanosarcina* within mangrove microbial consortia.

Using methanol or trimethylamine as the sole carbon substrate, four H_2_-independent methylotrophic methanogens, designed as T3, T4, T13, and MeOH, were successfully isolated from the magnetite-amended enrichments. Whole-genome sequencing revealed the genomes length of the four isolates ranging from 5.01 to 5.10 Mbp, comprising 152 to 157 contigs with N50 values between 70,734 and 75,521 bp (Table S2). All four genomes exhibited a completeness of 99.84% and 0% contamination, indicating their high quality and reliability. The 16S rRNA gene sequences of strains T3, T4, and T13 are identical, sharing 99.93% similarity with strain MeOH. The four isolates were most closely related to *M*. *siciliae* T4/M^T^, with 99.59–99.66% similarity in 16S rRNA gene sequence, 88.67–88.76% in ANI, 89.33–89.62% in AAI, and 35.6–35.7% in dDDH values (Table S2). Strains T3 and MeOH showed typical *Methanosarcina* morphology (Fig. S8). Phylogenetic analysis based on alignment of 53-archaeal marker proteins showed that the four isolates clustered with four *Methanosarcina* MAGs (Mes2, Mes3, Mes4, and Mes5), as well as with *M*. *siciliae* T4/M and *M. acetivorans* C2A (Fig. S9). However, the phylogenetic tree based on McrA sequences indicated that the four *Methanosarcina* isolates clustered with *Methanosarcina horonobensis* HB-1, rather than with constructed MAGs (Mes4, 5) or contigs assembled from the four metagenomic samples (Fig. S10)*.* These results suggest that *Methanosarcina* in the magnetite-amended groups comprised multiple species. The four isolates were closely related (Fig. S9) but not identical (Fig. S10) to the dominant *Methanosarcina* in magnetite-amended cultures.

After dereplication of the four *Methanosarcina* genomes with dRep, only the genome of *Methanosarcina* sp. T3 remained, which was used for further analysis. As a result, by integrating the sequencing data from metagenome and the genomes of in situ* Methanosarcina* isolates, 16 archaeal genomes were recovered (15 MAGs and 1 genome of *Methanosarcina* sp. T3), encompassing all methanogen types identified through 16S rRNA gene amplicon sequencing (Fig. 2).

### *Methanosarcina* enriched by magnetite addition belonged to Type II deficient with hydrogenotrophic methanogenesis

**Fig. 4:**
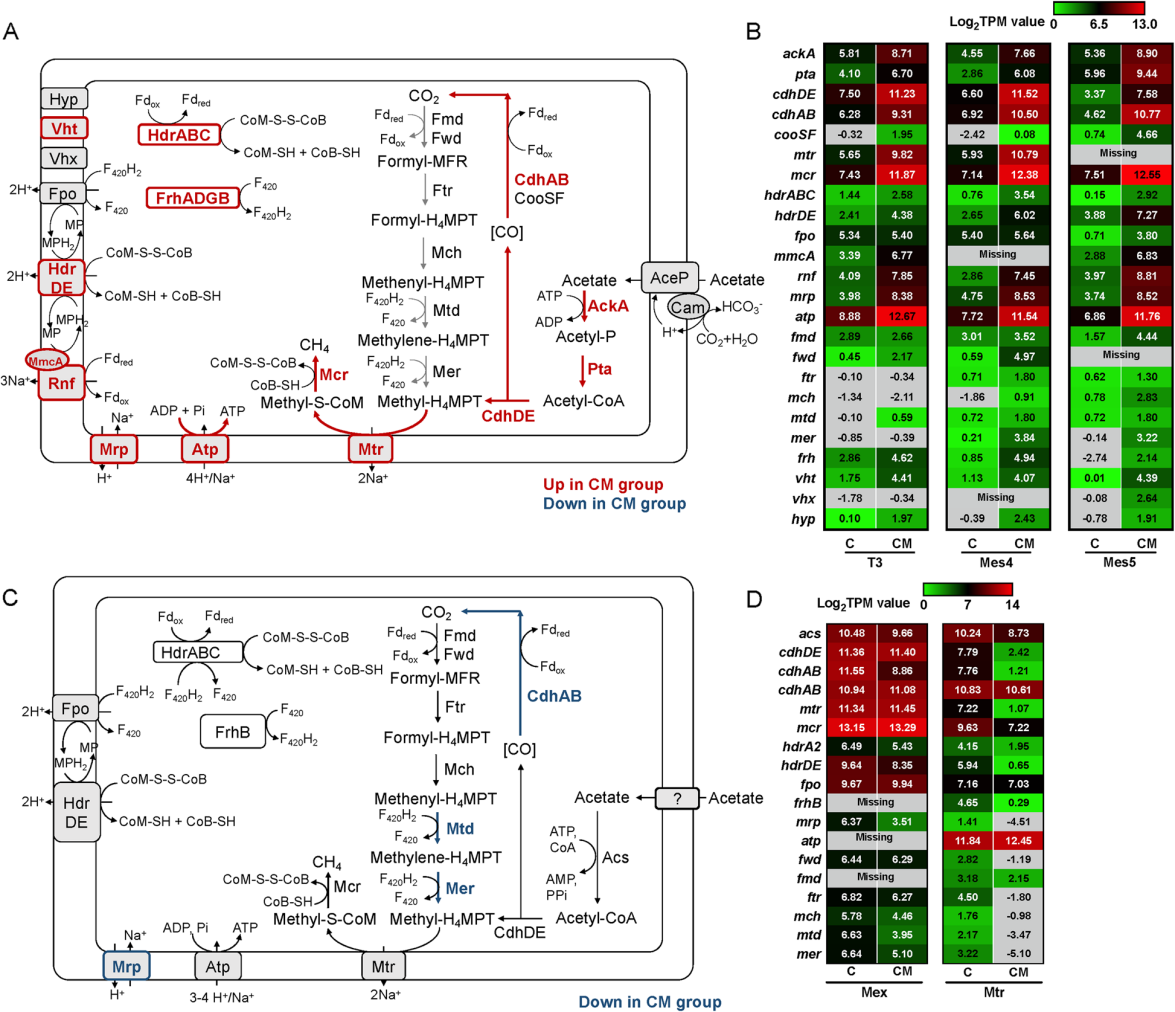
Aceticlastic methanogenesis pathways and transcript levels of the core genes in *Methanosarcina* (**A**, **B**) and *Methanotrichaceae* (**C**, **D**) in mangrove microbial consortia in the presence or absence of magnetite. For (**A**), genes that were significantly upregulated (log_2_FC > 1, FDR < 0.05) or downregulated (log_2_FC < − 1, FDR < 0.05) in magnetite-amended group for *Methanosarcina* sp. T3, Mes4 and Mes5 MAGs are presented in dark red and dark blue bold, respectively; for (**C**), genes that were significantly down-regulated in the control group (log_2_FC < − 1, FDR < 0.05) for both *Methanotrichaceae* Mex and Mtr MAGs are presented in dark blue bold; for (**B**) and (**D**), if proteins are composed of multiple subunits, values from the most highly expressed subunit are represented. *C* control group, *CM* magnetite-amended group. Details regarding the fold differences and FDR values of each gene are provided in Table S4

We further investigated the genomes of *Methanosarcina*, focusing primarily on the genome of *Methanosarcina* sp. T3 due to its high quality. As a typical *Methanosarcina*, strain T3 contains complete gene sets for aceticlastic methanogenesis and CO_2_ reduction (Fig. 4). Key genes for membrane-bound respiratory complexes including Rnf (Rhodobacter nitrogen fixation), heterodisulfide reductase HdrDE, F_420_ dehydrogenase Fpo, and multi-subunit sodium/proton antiporter Mrp are present, enabling Na^+^/H^+^ gradient formation for energy conservation [63, 64]. Additionally, strain T3 contains two cytoplasmic heterodisulfide reductases HdrABC and HdrA2B2C2 for reducing heterodisulfide CoB-S–S-CoM to CoB-SH and CoM-SH, along with ferredoxin oxidation [65]. Notably, strain T3 lacks genes coding for energy-converting Ech hydrogenase, which is essential for H_2_ metabolism in some *Methanosarcina* [18, 66]. Although strain T3 harbors several hydrogenase-related genes, including methanophenazine-reducing hydrogenase Vht/Vhx, hydrogenase maturation machinery Hyp, and F_420_-reducing hydrogenase FrhADGB, the attempt to grow strain T3 on H_2_/CO_2_ or formate was unsuccessful (Fig. S11). Thus, strain T3 was unable to perform hydrogenotrophic methanogenesis. Based on the above genomic and phenotypic evidence, we confirmed that strain T3 is a typical type II *Methanosarcina*.

As for *Methanosarcina* MAGs obtained by metagenome assembly, complete or partial Rnf complex genes were identified in four out of five MAGs, and *mmcA* gene was identified in Mes5 (Table S3). Notably, none of the *Methanosarcina* MAGs contains genes coding for Ech complex. Phylogenetic analyses using 53 archaeal marker proteins (Fig. S9) or McrA proteins (Fig. S10) indicated that the *Methanosarcina* MAGs clustered with type II *Methanosarcina* (*M*.* siciliae* and *M*. *acetivorans*). Thus, we proposed that *Methanosarcina* enriched in magnetite-amended microbial consortia are type II non-hydrogenotrophic.

### Metatranscriptome profiling of aceticlastic and hydrogenotrophic methanogens

Metatranscriptomic analyses were performed during the mid-logarithmic phase of methane production in the 5th generation in both magnetite-amended and control groups. Although bacteria accounted for more than half of the mapped reads in the metagenomic library, archaea were 4.8- and 3.5-fold more abundant in the metatranscriptomic library in the magnetite-amended and control groups, respectively (Fig. S12). And there was no significant difference in the relative abundance of bacteria between two groups (9.6% vs. 10.0%, *p* = 0.77) in metatranscriptomes. The results indicated that bacteria play only a minor role in the methanogenic process, and magnetite did not promote bacteria-methanogens interactions. Therefore, in this section, we primarily focused on aceticlastic and hydrogenotrophic methanogens that predominated in enrichments and evaluated their transcript response to magnetite addition. More discussions around effects of magnetite on bacterial MAGs are provided in supplementary materials.

### Aceticlastic methanogens

Similar to 16S rRNA gene amplicon sequencing (Fig. 2), metagenomic and metatranscriptomic analyses showed high activity of *Methanosarcina* in magnetite-amended group, while obligately aceticlastic *Methanotrichales* were more active in the control group (Fig. 3). The result verified that magnetite addition significantly altered the composition of aceticlastic methanogens.

In both magnetite-amended and control groups, strain T3 highly expressed genes for acetate dismutation, including *ackA*, *pta*, *cdhDE*, and *cdhAB* (log_2_TPM value > 4.1), while genes for CO_2_ reduction were merely expressed (log_2_TPM value < 2.9) (Fig. 4, Table S4). The transcriptional disparity suggested that strain T3 relied on acetate dismutation rather than CO_2_ reduction for methane production. Given that strain T3 cannot use hydrogen as an electron donor, this also indicated that extracellular electron transfer may not be crucial for its growth in magnetite-amended cultures. If *Methanosarcina* utilized extracellular electrons from Fe(II) in magnetite or from other microorganisms, genes for CO_2_ reduction would be correspondingly highly expressed [67].

Strain T3 upregulated genes for aceticlastic methanogenesis in the magnetite-amended group, including those for carbon metabolism (*ackA*, *pta*, *cdhDE*, *cdhAB*, *mtr*, and *mcr*) and energy conservation (*mmcA*, *rnf*, *mrp*) [63, 68] (Fig. 4, Table S4). Similar expression patterns were observed in *Methanosarcina* Mes4 and Mes5 MAGs (Fig. 4, Table S4). However, for acetate transporter, the gene (T3_1029) homolog to MA4008 (77.61% amino acid sequence identity) encoding acetate permease AceP [69] was significantly downregulated in magnetite-amended group (Log_2_FC = − 3.12). While another gene (T3_2034) encoding carbonic anhydrase Cam, predicated to form a complex with AecP for acetate transport (97.57% amino acid sequence identity with MA2536), was upregulated in the presence of magnetite (Log_2_FC = 2.07).

Conversely, *Methanotrichaceae* were more active in the control group (Fig. 3). We reconstructed the carbon metabolism and energy conservation pathway of two *Methanotrichaceae* MAGs Mex and Mtr (Fig. 4C). Notably, in Mex MAG, only *cdhAB*, *mtd*, *mer*, and *mrp* were more highly expressed in the control group, while genes coding for Acs, CdhDE, Mtr, Mcr, HdrDE, Fpo, or HdrABC did not show differential expression between groups. For another *Methanotrichaceae* MAG Mtr, although many genes for aceticlastic methanogenesis pathways were more highly expressed in the control group, some core genes like those encoding ATP synthase and Fpo, were not differently expressed (Fig. 4C).

### Hydrogenotrophic methanogens

**Fig. 5:**
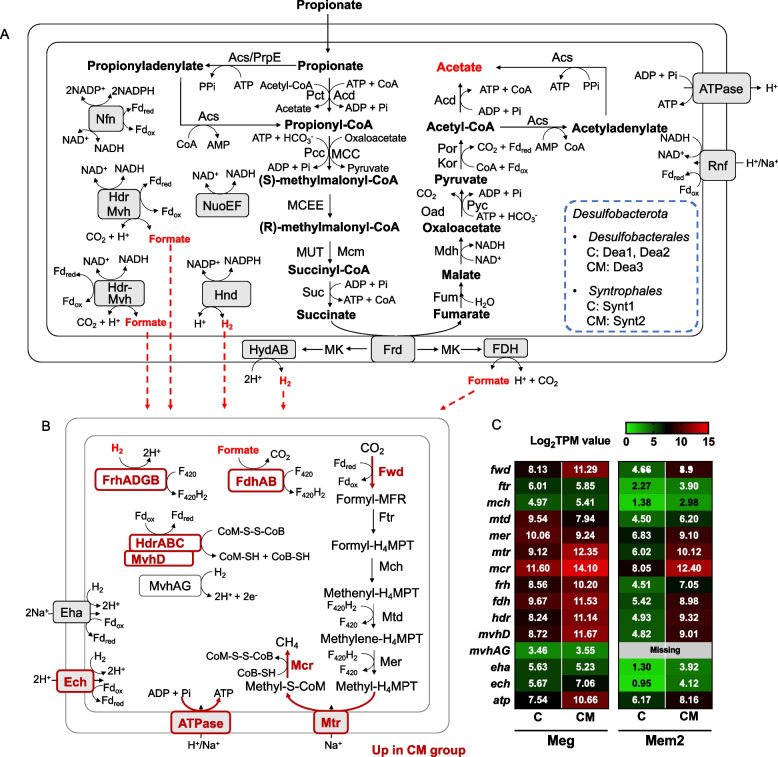
Hydrogenotrophic methanogenesis in *Methanomicrobiales* and syntrophic interactions. **A** Propionate degradation pathway of represented bacterial MAGs (blue dash line) supporting syntrophic interactions with hydrogenotrophic methanogens. **B** Hydrogenotrophic methanogenesis pathway in *Methanomicrobiales* MAGs (Meg and Mem2) during syntrophic growth with propionate-oxidizing bacteria. Genes up-regulated in the magnetite-amended group (log_2_FC > 1, FDR < 0.05) in both Meg and Mem2 are highlighted in bold dark red. **C** Transcript levels of core genes in *Methanomicrobiales* MAGs (Meg and Mem2). If proteins are composed of multiple subunits, values from the most highly expressed subunit are represented. *C* control group, *CM* magnetite-amended group. Fold differences and FDR values for each gene are provided in Table S4

Hydrogenotrophic methanogenesis plays an important role in our system, primarily supported by syntrophic interactions with propionate-oxidizing bacteria. Propionate oxidation is a thermodynamically unfavorable process under standard conditions, requiring syntrophic association with hydrogenotrophic methanogens to consume H_2_ (formate) to drive overall reaction forward [70]. Metatranscriptomic analysis revealed that members of the orders *Desulfobacterales* and *Syntrophales* within *Desulfobacterota* exhibited high transcriptional activities (> 1% relative abundances in the metatranscriptomic library, Fig. 3), indicating these two orders were primary syntrophic propionate-oxidizing bacteria. Dea3 (*Desulfobacterales*) and Synt2 (*Syntrophales*) were enriched in the magnetite-amended cultures, whereas Dea1, Dea2, and Synt1 were predominant in the control group. All of these MAGs harbored propionate oxidation pathways (Table S5), and key genes were highly expressed (Fig. S13, Fig. S14), confirming their active participation in propionate degradation. Despite these observations, we were unable to determine whether magnetite exerted selective pressure on specific syntrophic bacteria. Each of these taxa showed significantly higher expression of key functional genes in their respective groups compared to the other. Therefore, it remains unclear whether the enhanced expression was driven by magnetite or simply reflected differences in dominant taxa between the groups. Based on this, we focused our analysis on hydrogenotrophic methanogens to assess how magnetite modulates their methanogenic activity at the transcriptional level. Nevertheless, the overall pathway of bacterial propionate oxidation is also illustrated to provide a broader view of syntrophic interactions (Fig. 5).

At stage L in the 5th transfer, Meg (g_*Methanogenium*) was the most abundant hydrogenotrophic methanogen in both control and magnetite-amended groups, accounting for 6.2% and 8.3% of the total reads in metagenomic libraries, respectively. While addition of magnetite resulted in a 2.6-fold higher relative abundance of Meg in metatranscriptomic libraries (Fig. 3). Most of the core genes encoding hydrogenotrophic methanogenesis, including Fwd, Mtr, and Mcr were significantly upregulated in magnetite-amended groups, as well as membrane-bound energy-converting hydrogenases Ech and ATP synthase (Fig. 5). The coenzyme F_420_H_2_ cycle can be accomplished by coenzyme F_420_ hydrogenase (FrhADGB) or formate dehydrogenase (FdhAB), with hydrogen or formate as the electron donor, respectively. Genes coding for Frh and Fdh were also upregulated in magnetite-amended group. The CoB-S–S-CoM is reduced by cytoplasmic HdrABC, coupling to ferredoxin reduction via flavin-based electron bifurcation (FBEB). In hydrogenotrophic methanogen, F_420_-nonreducing hydrogenase (Mvh) provides reducing equivalents for Hdr through hydrogen oxidation [71–73]. But in our study, *mvhAG* were not differently expressed. A recent study has shown that FdhAB is able to oxidize formate coupled to Hdr in *Methanoculleus thermophilus* [74], within the same order *Methanomicrobiales* as Meg MAG. The highly expressed genes coding for FdhAB suggested that Meg utilized electrons from formate oxidation for Hdr complex as well.

Another hydrogenotrophic *Methanomicrobiaceae* Mem2 was also highly active in the magnetite-amended group, accounting for 1.4% and 2.6% of all reads in metagenomic and metatranscriptomic libraries, compared to 0.04% and 0.07% in the control group (Fig. 3). Mem2 upregulated all genes involved in hydrogenotrophic methanogenesis in magnetite-amended group, except that it lacks genes encoding MvhAG (Fig. 5, Table S4).

### Magnetite addition promoted aceticlastic methanogenesis of *Methanosarcina* sp. T3 grown in pure culture

**Fig. 6:**
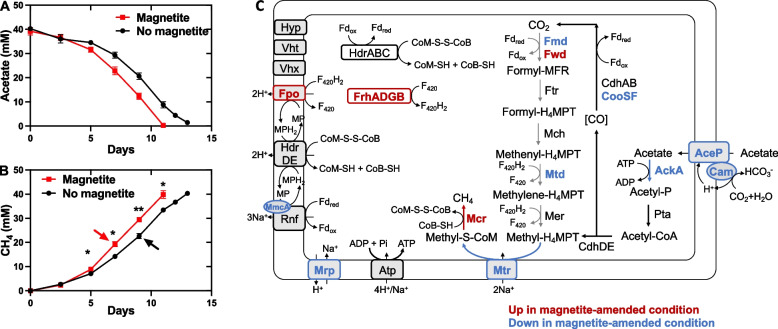
Effects of magnetite addition on growth (**A**, **B**) and transcript activity (**C**) of *Methanosarcina* sp. T3 in pure culture with acetate (40 mM) as the substrate. For (**A**) and (**B**), data represent mean values and standard deviations from three independent cultures. For (**C**), genes that were up-regulated and down-regulated in the presence of magnetite are presented in dark red and blue, respectively. Details regarding the fold differences and FDR values of each gene are provided in Table S6. Arrows indicated the sampling timepoints for transcriptomic analysis. Difference in methane production between the two groups were tested by two-tailed paired *t* tests, indicated by * when *p* < 0.05, or **p < 0.01

The effects of magnetite on type II *Methanosarcina* were evaluated using the pure culture of *Methanosarcina* sp. T3 with acetate (40 mM) as the sole substrate. The addition of magnetite significantly promoted aceticlastic methanogenesis of strain T3, resulting in 16.1 ± 2.6% higher production rate of methane production in the 5th generation (Fig. 6). Therefore, based on metatranscriptomic analysis and experimental validation, we confirmed that magnetite stimulated aceticlastic methanogenesis of type II *Methanosarcina* in microbial communities from mangrove sediments.

To explore the mechanisms by which magnetite promotes aceticlastic methanogenesis of type II *Methanosarcina*, we conducted transcriptomic analysis of *Methanosarcina* sp. T3 grown in pure culture under both magnetite-amended and non-magnetite conditions. Multi-dimensional scaling (MDS) revealed distinct separation in gene expression profiles of *Methanosarcina* sp. T3 in pure culture compared to that in mangrove microbial communities (Fig. S15). In the presence of magnetite, *Methanosarcina* sp. T3 upregulated 700 genes in pure culture, of which 218 genes were co-upregulated in mangrove microbial consortia. Conversely, a total of 678 genes were significantly down-regulated, with only 57 of those co-downregulated in mangrove consortia. These findings indicated the transcriptomic profiles of *Methanosarcina* sp. T3 in pure culture differ from those in mangrove microbial consortia, suggesting that the methanogenesis process in microbial consortia is much more complex than previously thought.

When grown in pure culture, *Methanosarcina* sp. T3 upregulated genes encoding Mcr complex in the presence of magnetite, consistent with the faster methane production rate (Fig. 6). Additionally, genes encoding the cytoplasmic Frh (*frhADG*, excluding *frhB*), membrane-bound Fpo (*fpoABCDHIJ1J2KLM*, excluding *frhN* and *frhO*), and Fwd (*fwdDBA*, excluding *fwdC*) were upregulated under magnetite conditions. However, none of these genes appear to be essential for acetate dismutation in type II *Methanosarcina*, as represented by *M*. *acetivorans* [75].

In contrast to genes upregulated in magnetite-amended mangrove consortia, many core genes for aceticlastic methanogenesis, such as *pta*, *cdhAB*, *cdhDE*, *rnf*, *hdrDE*, and *atp*, were not differentially expressed under pure culture conditions, despite their TPM values exceeding tenfold above the median level. Furthermore, genes encoding AceP, Mtr, AckA, Mrp, and multi-heme *c*-type cytochrome MmcA were even downregulated after magnetite addition (Fig. 6, Table S6). In particular, *cam* encoding carbonic anhydrase related to acetate transport was among the most down-regulated genes in magnetite-amended pure culture (Log_2_FC = − 3.5). Although its physiological function remains uncharacterized, Cam contains iron in its active site [76], which could be influenced by magnetite.

An intriguing observation was that the addition of magnetite caused differential expression of genes encoding various surface-associated proteins (Table S6). For example, strain T3 has six genes encoding archaeal type IV pilus assembly protein PilA, five of which were significantly up-regulated in the presence of magnetite (Log_2_FC > 1.65). Genes encoding archaeal flagellin structure protein FlaB and the accessory flagellar proteins FlaGFHIJ were among the most upregulated genes (Log_2_FC > 5.6) under magnetite-amended conditions. Additionally, genes related to disaggregatase-associated membrane protein (T3_2961) and extracellular matrix structural constituent (T3_1080) were significantly upregulated (Log_2_FC > 3.4) under magnetite conditions in both pure culture and mangrove consortia. A gene cluster comprising five genes encoding hypothetical proteins was identified in the genome of strain T3 (T3_3948-3952) and was significantly upregulated (Log_2_FC > 5.9) in the presence of magnetite under pure culture conditions. The function of this gene cluster was unknown, but three genes were predicted to be non-cytoplasmic and possess a transmembrane helix and/or signal peptide (Table S7). Conversely, two genes encoding S-layer protein (T3_422, T3_3753) were significantly downregulated under magnetite-added condition (Log_2_FC < −4.4).

## Discussions

Magnetite, a widely occurring iron oxide mineral, has been shown to facilitate microbial methanogenesis in natural and artificial environments [77]. In this study, we systematically investigated the effects of magnetite on the methanogenesis of microbial communities from mangrove sediments. Magnetite stimulated methane production and reshaped the community structure of mangrove microbial consortia. However, unlike previous studies focusing primarily on interactions between bacteria and methanogens [27, 78], our results underscore the direct stimulatory effects of magnetite on methanogens themselves. Especially, by integrating multi-omics analyses with experimental validation, we demonstrate that magnetite addition promotes aceticlastic methanogenesis of type II *Methanosarcina*.

Type II *Methanosarcina*, represented by *M*. *acetivorans*, are non-hydrogenotrophic methanogens lacking the Ech complex, but use Rnf complex for energy conservation, and outer-surface *c*-type cytochrome MmcA provides these methanogens additional capacity for extracellular electron transfer with environments, including Fe(III), F(0), AQDS and other microorganisms [17, 67, 79, 80]. In contrast, type I hydrogenotrophic *Methanosarcina*, represented by *M*. *barkeri*, lack Rnf complex or MmcA, relying on intracellular H_2_ cycling through Ech complex to conserve energy [18, 66]. Besides physiological dichotomy, the preferred habitats of the two groups of *Methanosarcina* are distinct: type I *Methanosarcina* are well suited for organic-rich anaerobic digesters. While type II *Methanosarcina* thrived in organic-poor soil and sediment environments, and may have a more significant ecological impact on global methane emissions compared to type I *Methanosarcina* [17].

Recent studies have reported that magnetite enhances aceticlastic methanogenesis of type I hydrogenotrophic *Methanosarcina* including *M*. *barkeri* and *M*. *mazei* [24, 25]. This enhancement may be linked to magnetite’s redox behavior, which facilitates intracellular and/or membrane electron transfer during aceticlastic methanogenesis. Type I *Methanosarcina* were frequently enriched under magnetite conditions in environmental samples [25, 81]. These results bring us to another question: why were type II *Methanosarcina* enriched in the magnetite-amended group, not type I hydrogenotrophic *Methanosarcina*? An ecological fact is that the inoculum diversity may influence this enrichment process. However, mangrove sediments being organically rich environments, are favorable for Type I *Methanosarcina* compared to ecosystems such as soil and freshwater [17]. Thus, the co-occurrence of both types in the sediment inoculum is expected. Excluding inoculum bias, two factors may account for the enrichment of type II *Methanosarcina* in the magnetite-amended group: (1) metatranscriptomic data indicated that strictly hydrogenotrophic *Methanomicrobiales* were more activated in condition of magnetite addition, which display higher hydrogen affinities and growth rates than type I *Methanosarcina* [9]. In such conditions, type I hydrogenotrophic *Methanosarcina* was less competitive for hydrogen than *Methanomicrobiales*; (2) type II *Methanosarcina* assembled with Rnf complex and MmcA exhibited stronger positive response to magnetite than type I *Methanosarcina* assembled with Ech complex.

Interestingly, unlike previous studies using acetate as the sole substrate in defined systems [24], we observed that magnetite addition did not significantly shorten the lag phase of methane production in our enrichment cultures (Fig. 1). This discrepancy may be attributed to the complex metabolite interactions in our system, where acetate was not the only available substrate, but a secondary metabolite derived from lactate and propionate fermentation. In such a multi-step microbial food chain, the onset of aceticlastic methanogenesis is dependent not only on *Methanosarcina* activity, but also on upstream fermenters and syntrophic propionate-oxidizers. Therefore, even though magnetite may enhance the activity of aceticlastic methanogens at stage L, its effect on the overall lag phase of methane production might be masked or delayed by rate-limiting steps in the preceding fermentative or syntrophic pathways. This highlights the importance of ecological context when evaluating magnetite’s stimulatory effects in complex microbial communities.

To our surprise, transcriptomic analysis of *Methanosarcina* sp. T3 grown in pure culture did not reveal a significant metabolic pattern compared with that in microbial consortia. *Methanosarcina* possesses efficient electron transport systems, using methanophenazine (MPH) as the membrane electron carrier for energy conservation. Previous studies showed that magnetite can enhance electron transfer in type I *Methanosarcina*, by establishing an electrical connection with outer-surface *c*-type cytochromes for *M*. *mazei* [25], or by acting as the electron carrier similar to MPH for *M*. *barkeri* [24]. But in our study, genes involved in electron transfer chain of *Methanosarcina* sp. T3, for example *rnf* and *mmcA*, were not corresponding upregulated in pure culture under magnetite-adding condition. In fact, compared with type I *Methanosarcina*, type II *Methanosarcina* has more electron carrier MPH in its membrane and generates higher electron flux through MPH for energy conservation [82]. Taken together with the lack of differential expression of membrane-bound redox proteins, these findings suggest that magnetite may stimulate aceticlastic methanogenesis in type II *Methanosarcina* via mechanisms distinct from those described for type I *Methanosarcina*.

Although the underlying mechanism remains unclear, one defined phenomenon was the significant upregulation of genes encoding archaeal flagellin FlaB under magnetite-amended condition, both in pure culture (T3_3007, log_2_FC = 6.0) and in microbial consortia (T3_3026 and T3_3027, log_2_FC = 2.1 and 2.7, respectively) (Table S4, S5). Archaella are essential for type II *Methanosarcina M*. *acetivorans* to direct accept electrons from *Geobacter*, and the high aromatic amino acid content of archaellins suggests their potential to form electrically conductive archaella [67]. Strain T3 codes three putative archaeal flagellins FlaB (T3_3007, T3_3026, and T3_3027). However, none of them meet the criterion as conductive pili/archaella (> 9% aromatic amino acids, largest aromatic-free gap < 40 amino acids, Fig. S16) [83, 84]. This observation raises the possibility that magnetite may stimulate aceticlastic methanogenesis in type II *Methanosarcina* through alternative mechanisms. Considering the significant differential expression of multiple membrane-associated proteins (Table S6), one hypothesis is that magnetite enhances redox buffering or facilitates cell-particle interactions that improve intracellular electron transfer efficiency or substrate acquisition. Alternatively, the differential regulation of FlaB and other membrane proteins may reflect increased cellular motility or aggregation behavior in response to magnetite, which may indirectly enhance metabolic efficiency. Future studies involving *flaB* gene knockout, electrochemical assays, and microscopy-based cell-mineral interaction analyses may help validate the hypotheses.

Another interesting observation is that magnetite addition caused sharp decrease for relative abundance of *Methanothrix* within family *Methanotrichaceae*. *Methanosarcina*, with its faster growth rate and metabolic versatility, would typically outcompete *Methanothrix* under high acetate conditions. The initial dominance of *Methanosarcina* in the 1 st generation likely reflects its rapid response to elevated acetate (~ mM range) supplied via lactate degradation (Fig. 2). However, *Methanothrix* prevailed in the 3rd and 5th generations, likely due to its higher acetate affinity (5–20 μM), which allowed it to exploit locally lower or fluctuating acetate levels shaped by syntrophic bacteria (*Desulfobacterales*, *Syntrophales*). Previous study indicated that magnetite addition has been proved to promote the growth and methane production of *Mx*. *thermoacetophila* during aceticlastic methanogenesis as well as DIET with *Geobacter* [26]. High relative abundance and transcriptomic activity of *Methanothrix* were also observed in many magnetite-amended anaerobic digesters [85, 86]. In our study, despite the lower metagenomic and metatranscriptomic abundances of *Methanotrichaceae* (Fig. 3), many core genes coding for carbon metabolism and energy conservation were not corresponding down-regulated in the magnetite-amended group (Fig. 4, Table S4). Therefore, we suggest that this phenomenon is not due to the inhibition of aceticlastic methanogenesis activity in *Methanotrichaceae* by magnetite, but rather because *Methanosarcina* shows a stronger positive response to magnetite than *Methanothrix.* This stimulatory effect for *Methanosarcina* accumulated along with serial transfers, resulting in differences in relative abundances. Besides, this study also highlights the ecological significance of *Methanotrichaceae* in mangrove sediments, considering their predominant role in the control group.

Hydrogen is a common bioproduct in lactate metabolism for bacteria, and hydrogenotrophic methanogenesis occupied non-ignorable roles after aceticlastic methanogenesis in this study. The majority of hydrogenotrophic methanogens enriched in two groups at 5th generation belong to the order *Methanomicrobiales* (Fig. 3, Fig. S10). *Methanomicrobiales* are obligately hydrogenotrophic methanogens with higher H_2_ affinity than versatile *Methanosarcina*, making them a strong H_2_-consuming competitor in natural environments [9]. Previous study has shown that *Methanomicrobiales* is one of the most abundant and activated methanogens in marine ecosystems including mangrove sediments, which we used as inoculum [87], and magnetite addition further increased their transcriptional activity (Fig. 5). However, *Methanomicrobiales* lack membrane-associated *c*-type cytochrome or methanophenazine [9], which are predicted to interact with or be replaced by magnetite as an electron shuttle to promote aceticlastic methanogenesis of *Methanosarcina* [24, 25]. A previous study showed that magnetite did not stimulate the hydrogenotrophic methanogenesis of *Methanococcus maripaludis* or *Methanocella conradii*, two genera within the order *Methanocellales*, but promoted their syntrophy with *Syntrophomonas* [78]. In the present study, the underlying mechanisms by which magnetite interacts with *Methanomicrobiales* to promote hydrogenotrophic methanogenesis remain unknown, thus more experimental verifications need to be done using pure culture of *Methanomicrobiales* strains.

Another interesting observation was the consistent detection of *Candidatus* Methanofastidiosales across all serial transfers especially in the control group. *Candidatus* Methanofastidiosales is an uncultivated archaeal lineage frequently detected in various methanogenic environments including the mangrove sediments that used as inoculum in this study [87]. Previous researches indicated that this lineage lacks genes for Wood-Ljungdahl pathway, but relies on methyltransferases to produce methane from methyl compounds [88, 89]. In this study, two MAGs Mef1 and Mef2 (88.85–89.45% completeness, 2.16–2.65% contamination) were recovered from the enrichments, and their proposed carbon metabolism and energy conservation pathway were reconstructed (Fig. S17, Table S8). Potential routes for *Methanofastidiosales* to acquire carbon are presented in the supplementary text.

## Conclusion

The addition of magnetite enhanced methanogenesis in microbial consortia from mangrove sediments over five serial transfers using lactate as the substrate. By integrating results of metabolic profiling, 16S rRNA gene amplicon sequencing, metagenome and metatranscriptome analyses, as well as strain isolation and validation, we confirmed that the addition of magnetite stimulated the enrichments of type II non-hydrogenotrophic *Methanosarcina* and promoted their aceticlastic methanogenesis. Furthermore, metatranscriptomic analysis suggested that magnetite also enhanced hydrogenotrophic methanogenesis of *Methanomicrobiales* in the mangrove consortia. The results offer a comprehensive insight into the complex interactions between magnetite and diverse methanogens in natural microbial communities, and provide a deeper understanding of their physiology of type II *Methanosarcina*.

## Supplementary Information

Supplementary Material 1.

Supplementary Material 2.

Supplementary Material 3.

Supplementary Material 4.

## Data Availability

The 16S rRNA gene amplicon, metagenomic, metatranscriptomic, and transcriptomic data have been deposited in the Genome Sequence Archive (GSA: https://ngdc.cncb.ac.cn/gsa/) in National Genomics Data Center under BioProject accession number PRJCA037270 (CRA023792, CRA023773, CRA023785, CRA023795), and in the NODE database (https://www.biosino.org/node) under accession numbers OEP00005948 and OEP00005956. The whole genome sequence data including 117 MAGs and 4 genomes of *Methanosarcina* isolates have been deposited in the Genome Warehouse (GWH: https://ngdc.cncb.ac.cn/gwh/) in National Genomics Data Center under accession numbers GWHFQOD00000000-GWHFQOZ00000000, GWHFQPA00000000-GWHFQPZ00000000, GWHFQQA00000000-GWHFQQZ00000000, GWHFQRA00000000-GWHFQRZ00000000, and GWHFQSA00000000-GWHFQST00000000.
