# Supplementary material for: Magnetite drives microbial community restructuring and stimulates aceticlastic methanogenesis of type II Methanosarcina in mangrove sediments
Source: Microbiome. 2025 Jul 26;13:174. doi: 10.1186/s40168-025-02157-z (PMC12297640; doi:10.1186/s40168-025-02157-z)
Supplement: Supplementary file 2 — Supplementary Material 1. [file 40168_2025_2157_MOESM1_ESM.pdf]

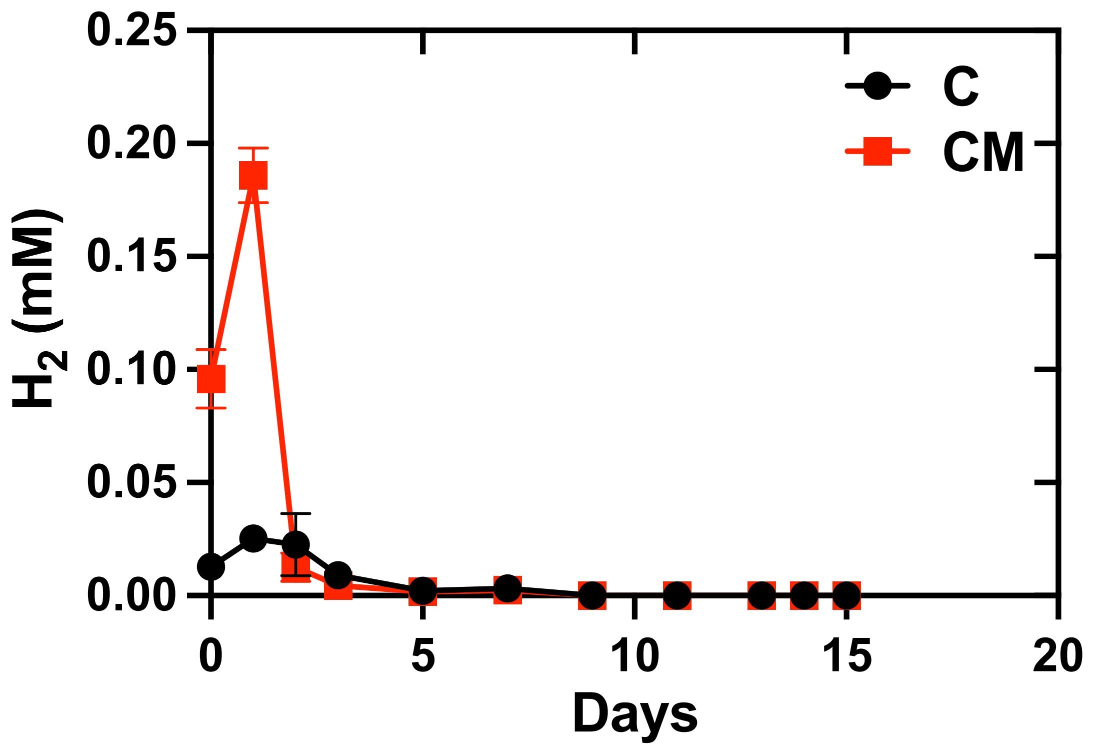

Figure S1. H_2 production of microbial consortia in the control group (C) and magnetite-amended group (CM) at the 5th generation. Data represent mean values and standard deviations from three independent cultures.

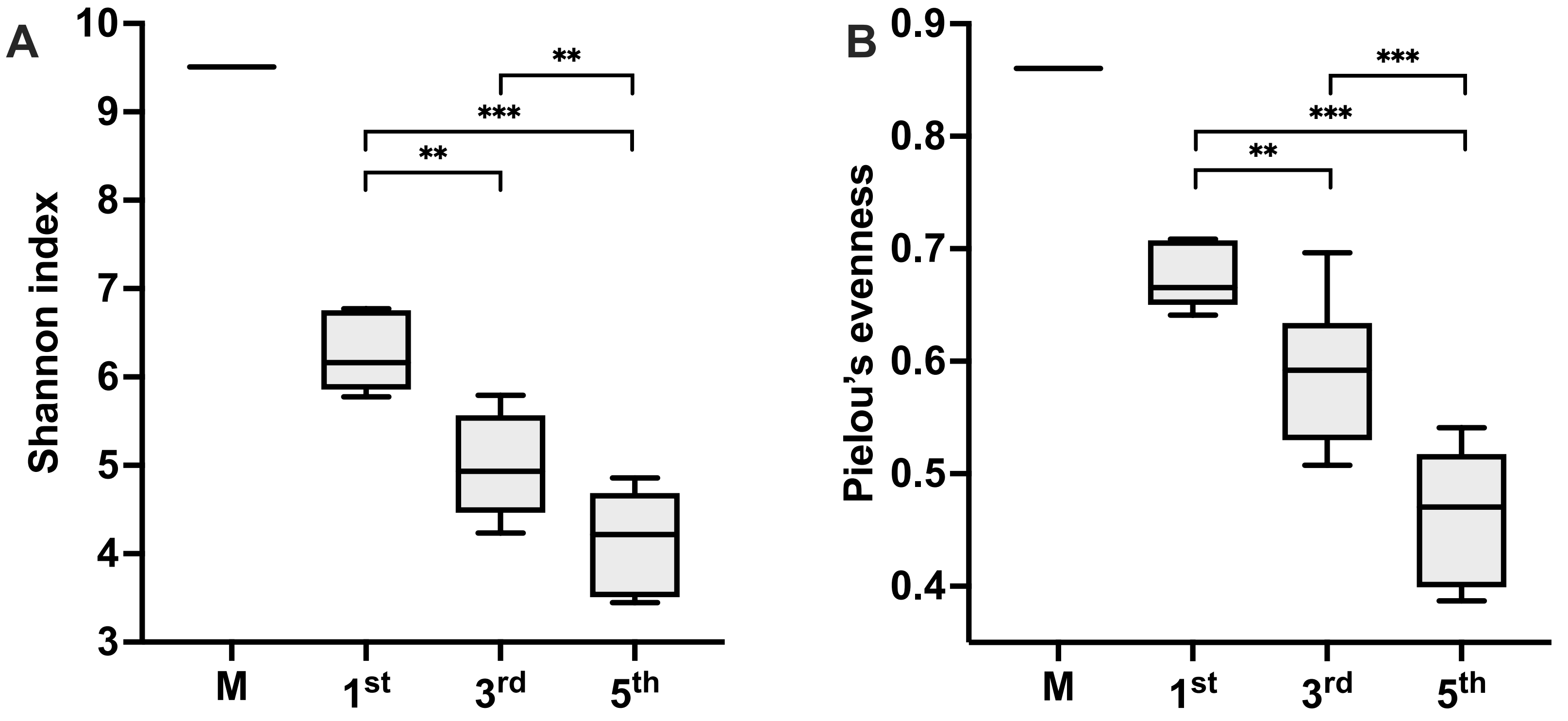

Figure S2. Alpha diversity indices of mangrove microbial communities during serial transfers. (A) Shannon index, (B) Pielou's evenness. The x-axis represents the transfer generations (1st, 3rd, and 5th) and the original inoculum (M, n=1). Each boxplot combined data from both control (C) and magnetite-amended (CM) groups across stages E and L within the same generation. For the 1st generation, n = 6 (3 replicates × 2 groups), and for the 3rd and 5th generations, n = 12 per generation (3 replicates × 2 groups × 2 stages). Statistical significance among groups was determined by QIIME2 using Kruskal-Wallis (pairwise) method. Significance levels: *$p < 0.05$, **$p < 0.01$, ***$p < 0.001$.

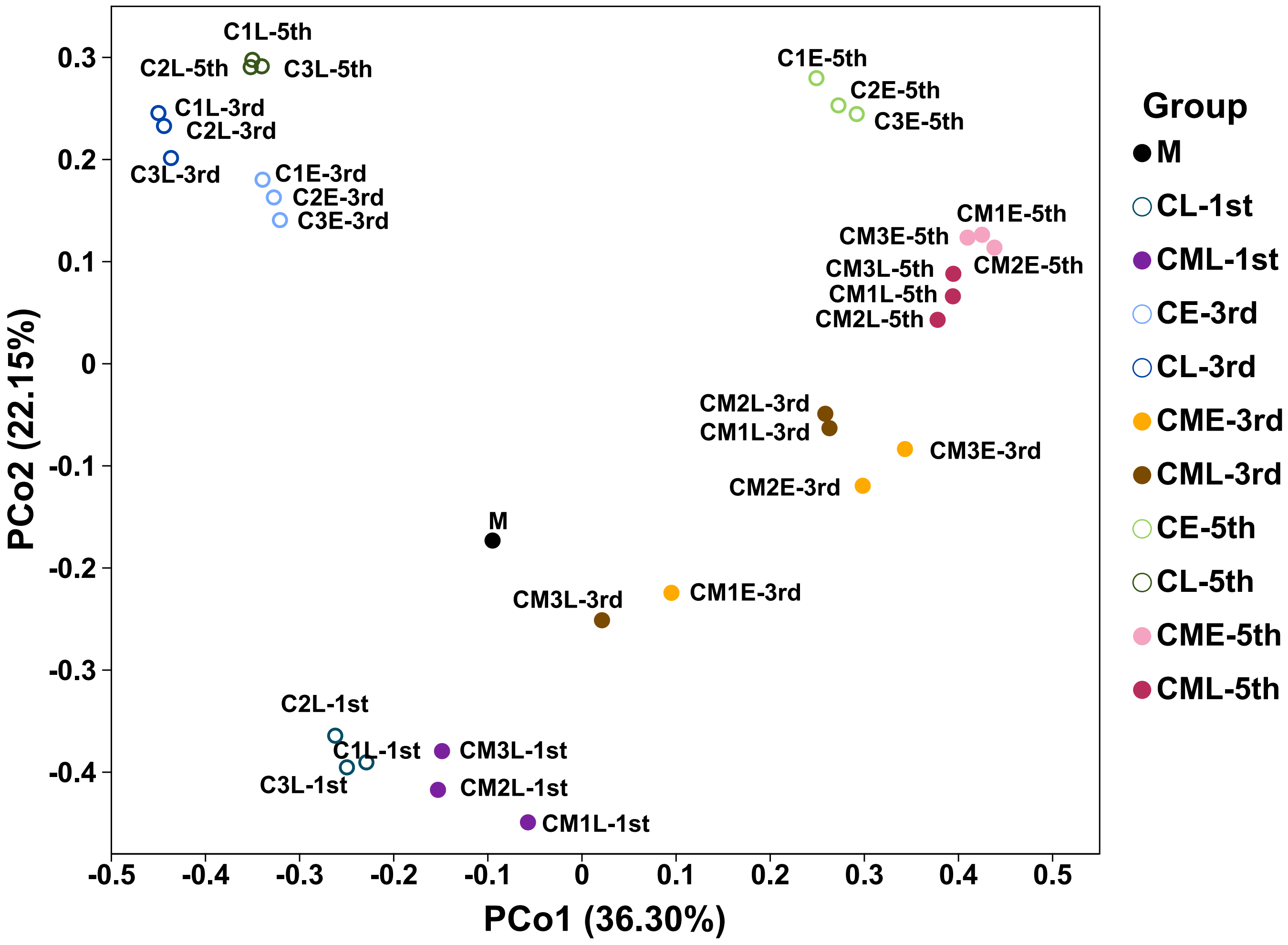

Figure S3. PCoA plot analysis of microbial communities in 1st, 3rd, and 5th generations in the control group (C) and magnetite-amended group (CM) at stage E and stage L. M: inoculum.

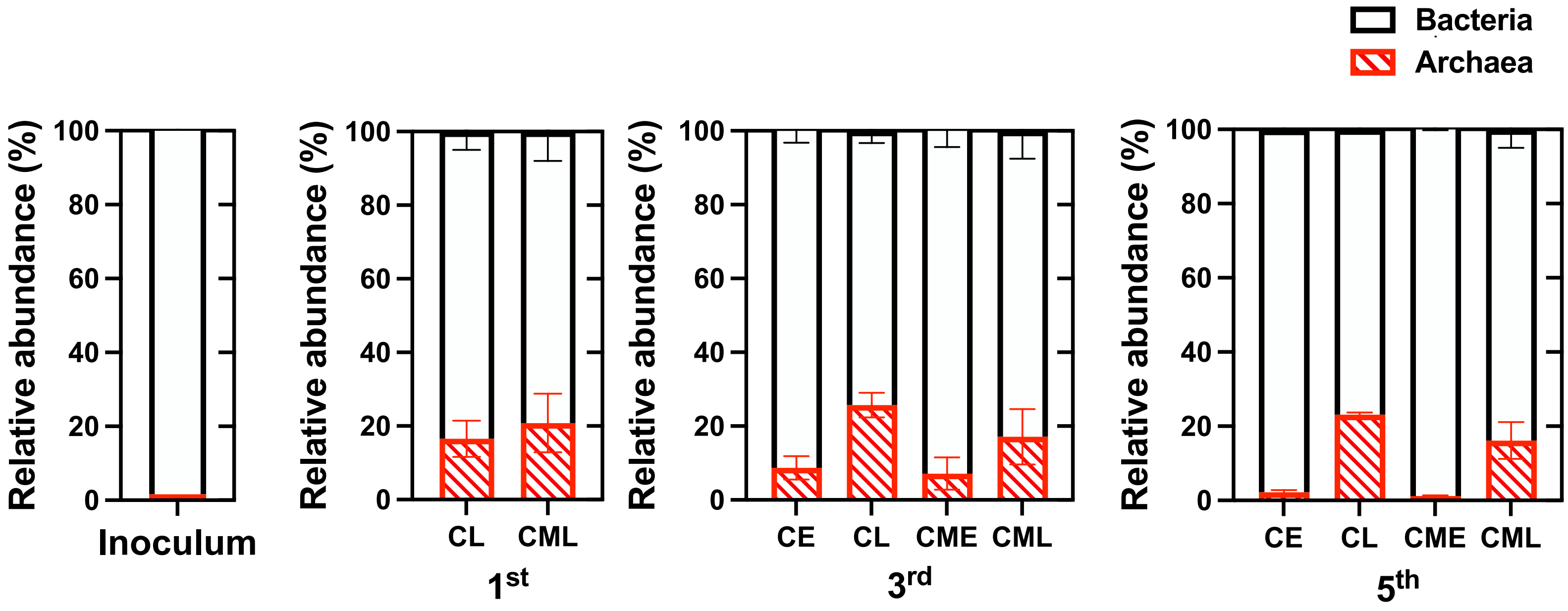

Figure S4. Relative abundance of bacteria and archaea on domain level in the inoculum, 1st, 3rd and 5th transfer in the control group (C) and magnetite-amended group (CM) at stage E and stage L. Bar represents the means of values obtained from three independent cultures.

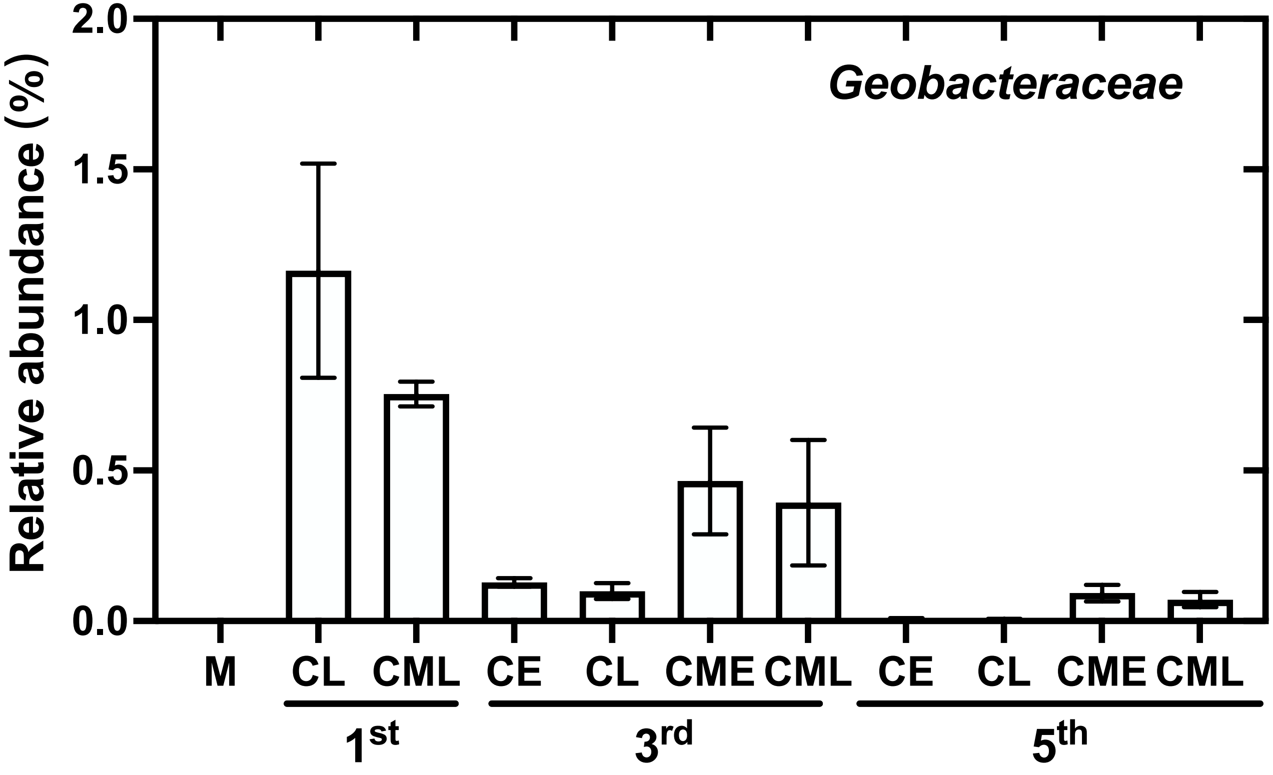

Figure S5. Relative abundance of *Geobacteraceae* family of the total bacteria in microbial communities at 1st, 3rd and 5th generations. CE and CL, stage E and L for the control group; CME and CML, stage E and L for the magnetite-amended group. Bar represents the means of values obtained from three independent cultures. M: inoculum.

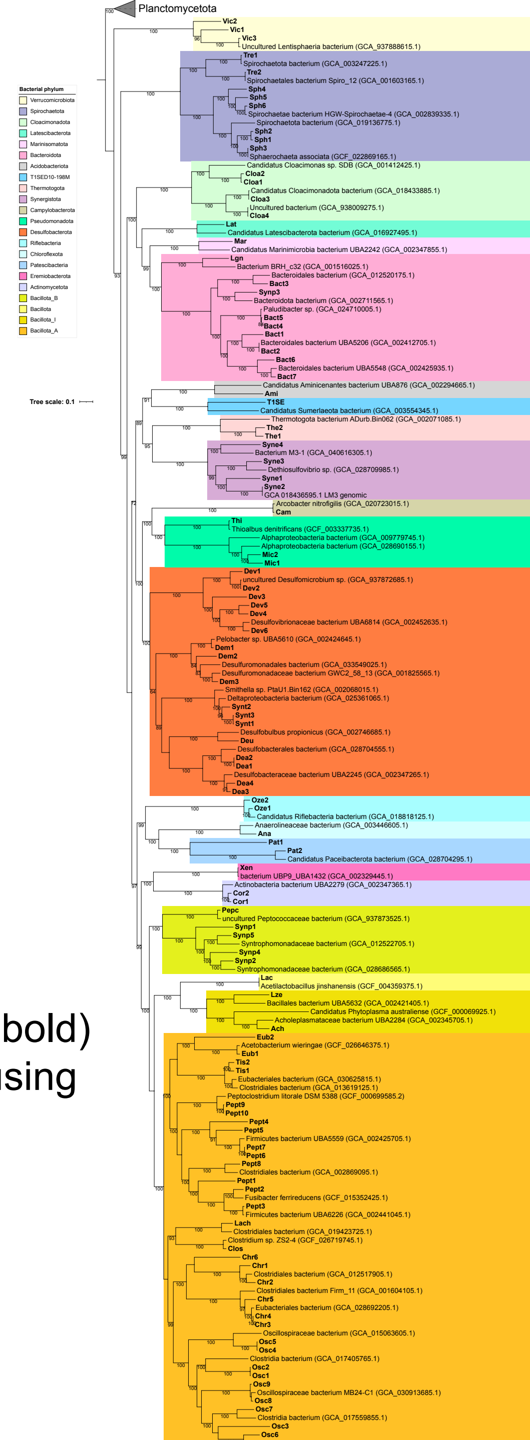

Figure S6. Phylogenetic tree of bacterial MAGs recovered from this study (in bold) and the relatives based on 120 bacterial single-copy marker proteins using Planctomycetota as the outgroup.

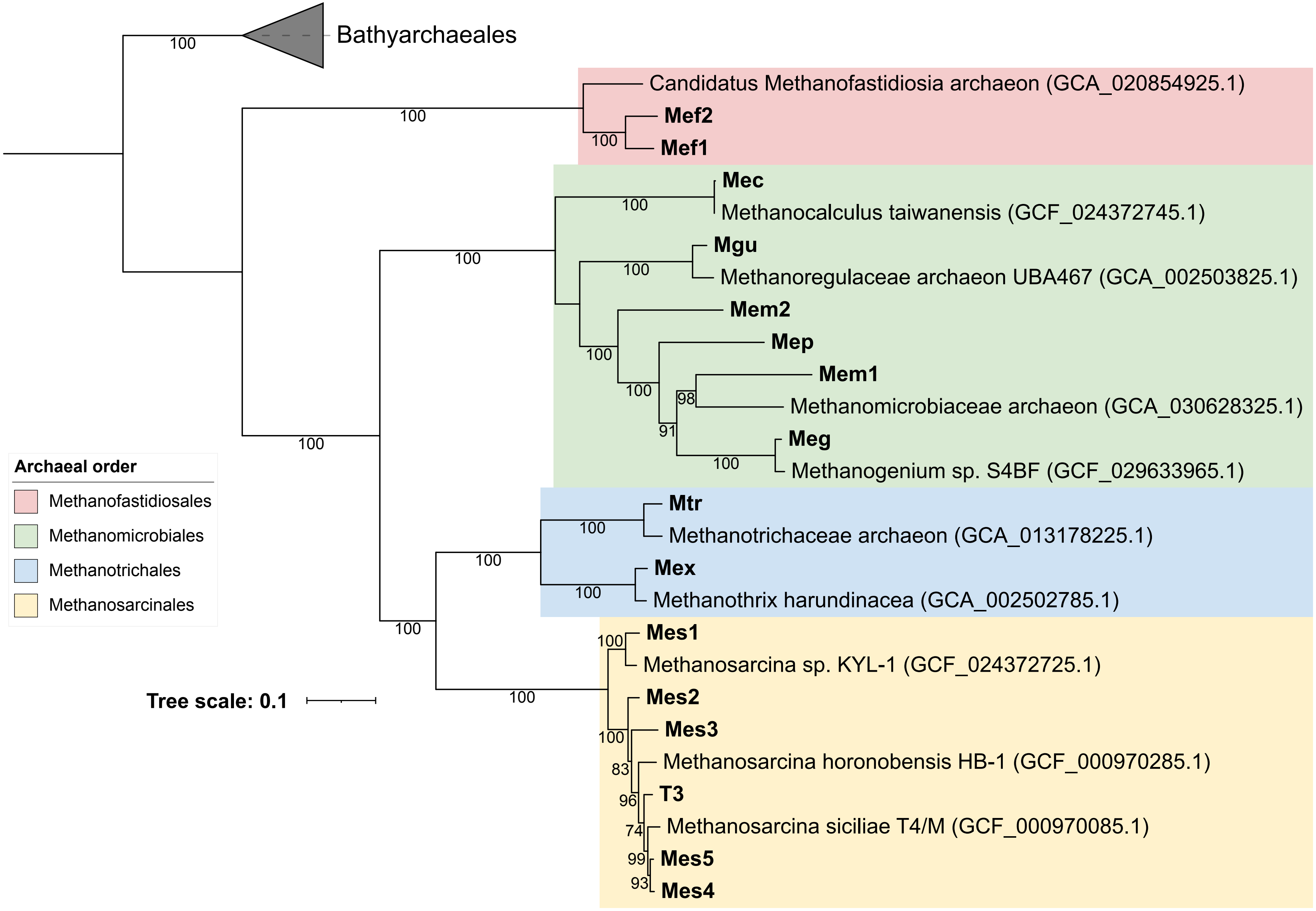

Figure S7. Phylogenetic tree of archaea genomes recovered from this study (15 MAGs and 1 genome of strain of T3, in bold) and the relatives based on 53 archaeal single-copy marker proteins using Bathyarchaeales as the outgroup.

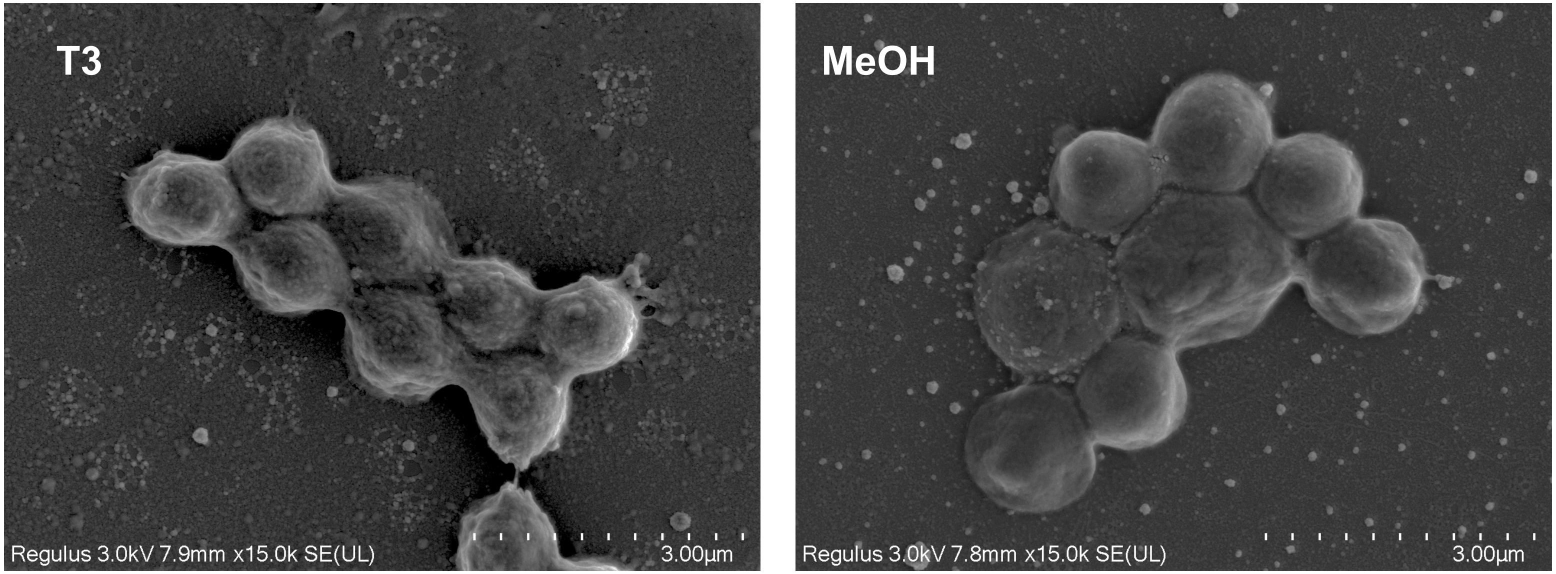

Figure S8. Scanning electron micrographs of *Methanosarcina* sp. T3 and MeOH.

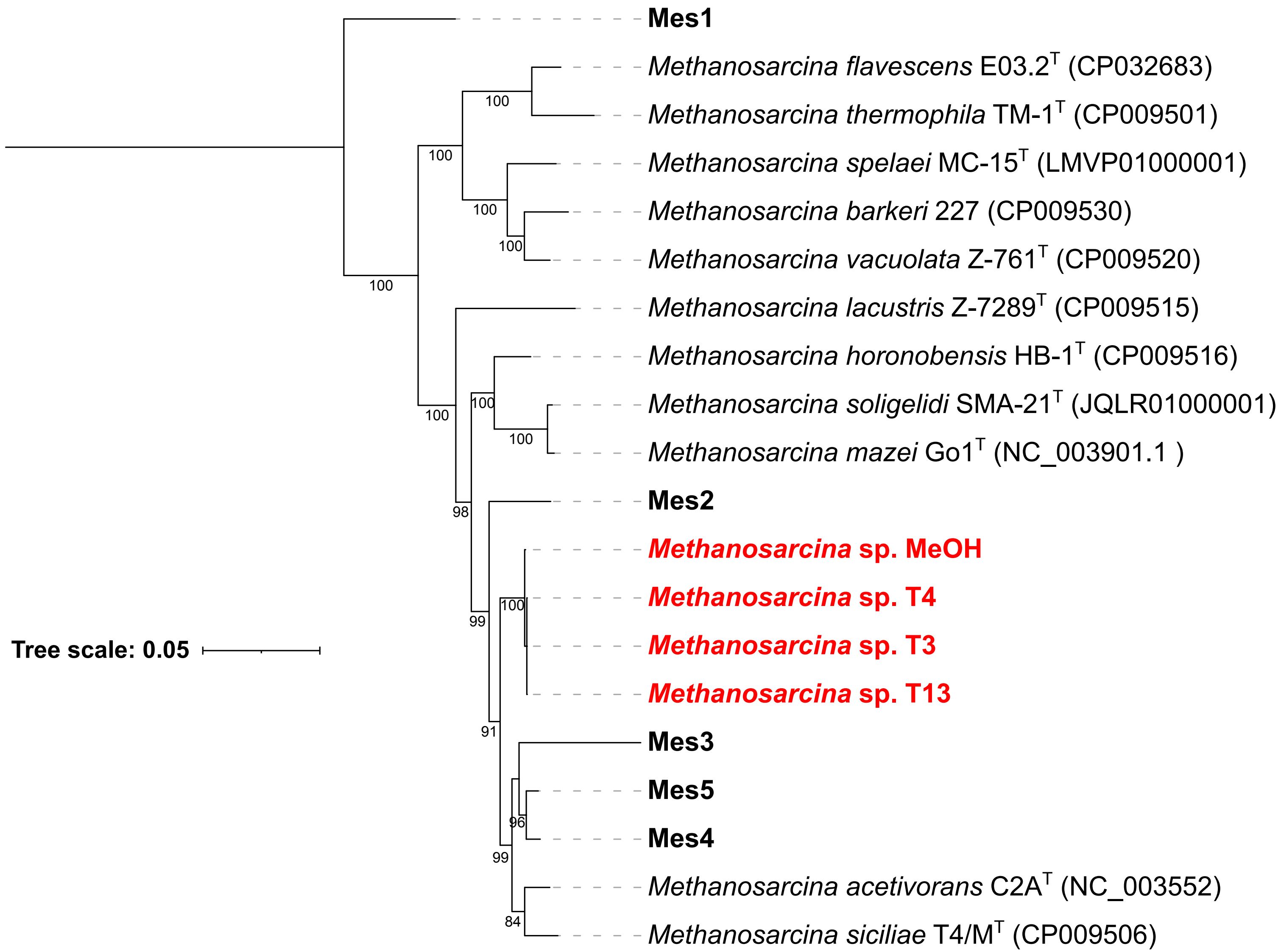

Figure S9. Phylogenic tree of *Methanosarcina* MAGs and isolates based on 53 archaeal marker proteins provided by GTDB-Tk. Genome of *Methanococcoides methylutens* MM1 (CP009518) was used as the outgroup (not shown).

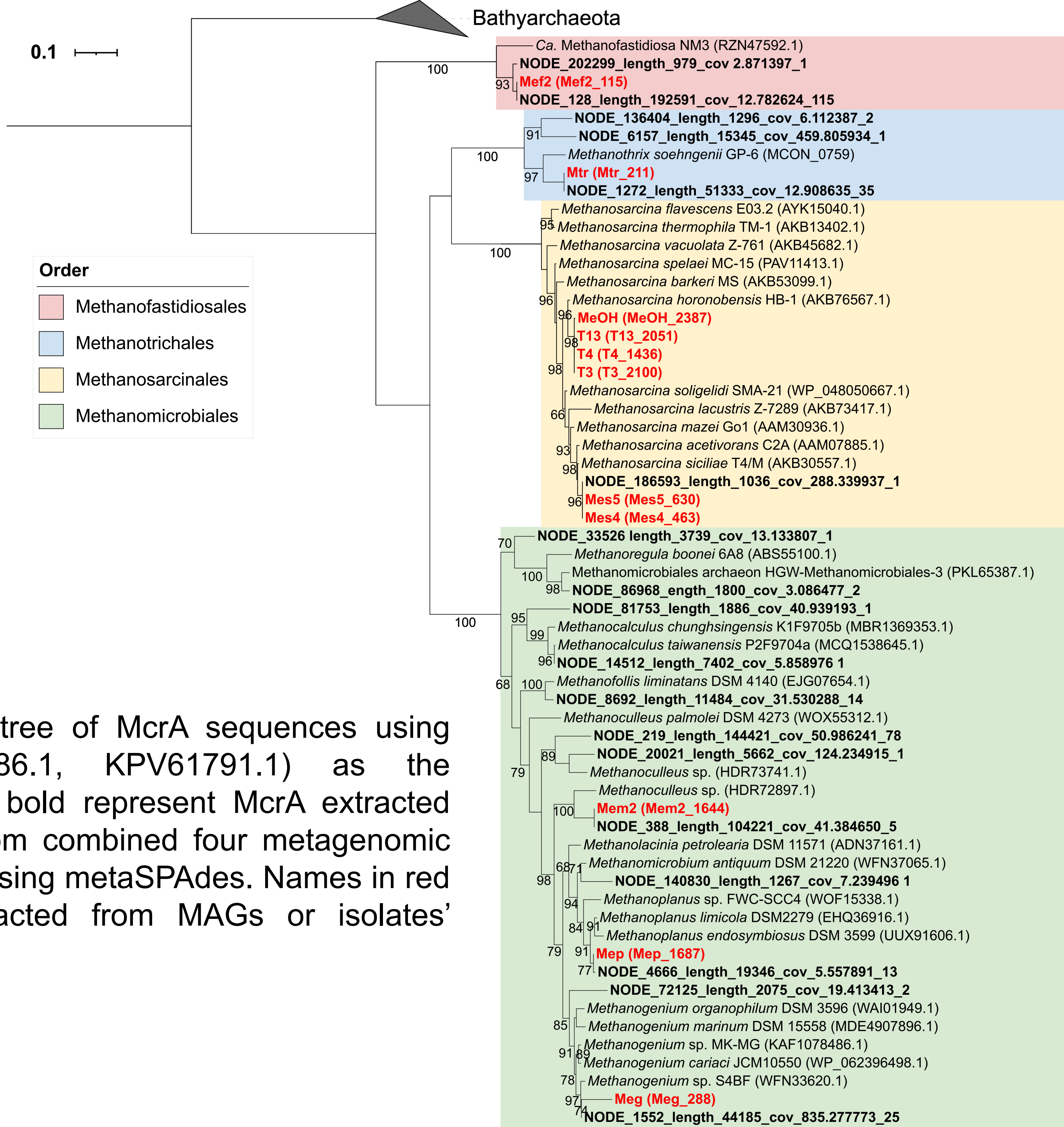

Figure S10. Phylogenetic tree of McrA sequences using Bathyarchaeota (KPV65186.1, KPV61791.1) as the outgroup. Names in black bold represent McrA extracted from contigs assembled from combined four metagenomic data (CE, CL, CME, CML) using metaSPAdes. Names in red bold represent McrA extracted from MAGs or isolates' genomes.

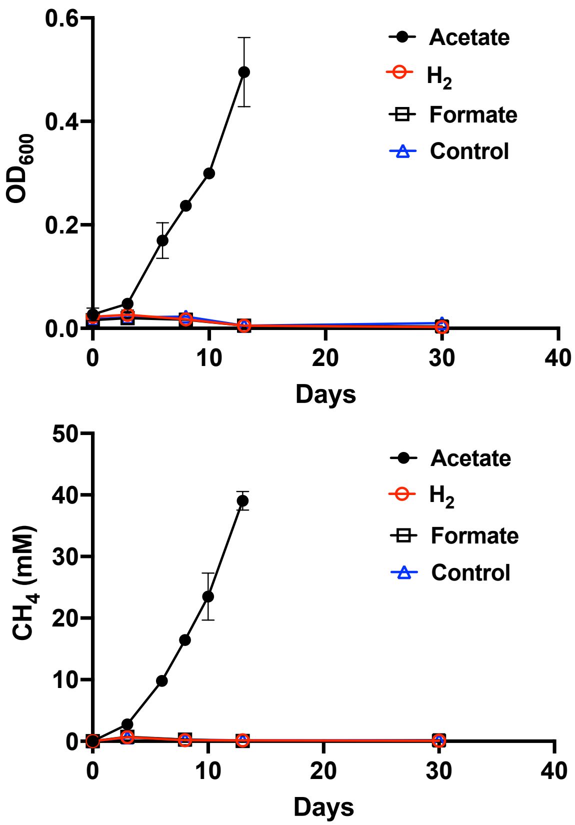

Figure S11. Growth and methane production of *Methanosarcina* sp. T3 using H_2 (H_2/CO_2, 80/20), formate (40 mM), and acetate (40 mM) as substrates. Cultures without substrate addition were used as control. Data represent mean values and standard deviations from three independent cultures.

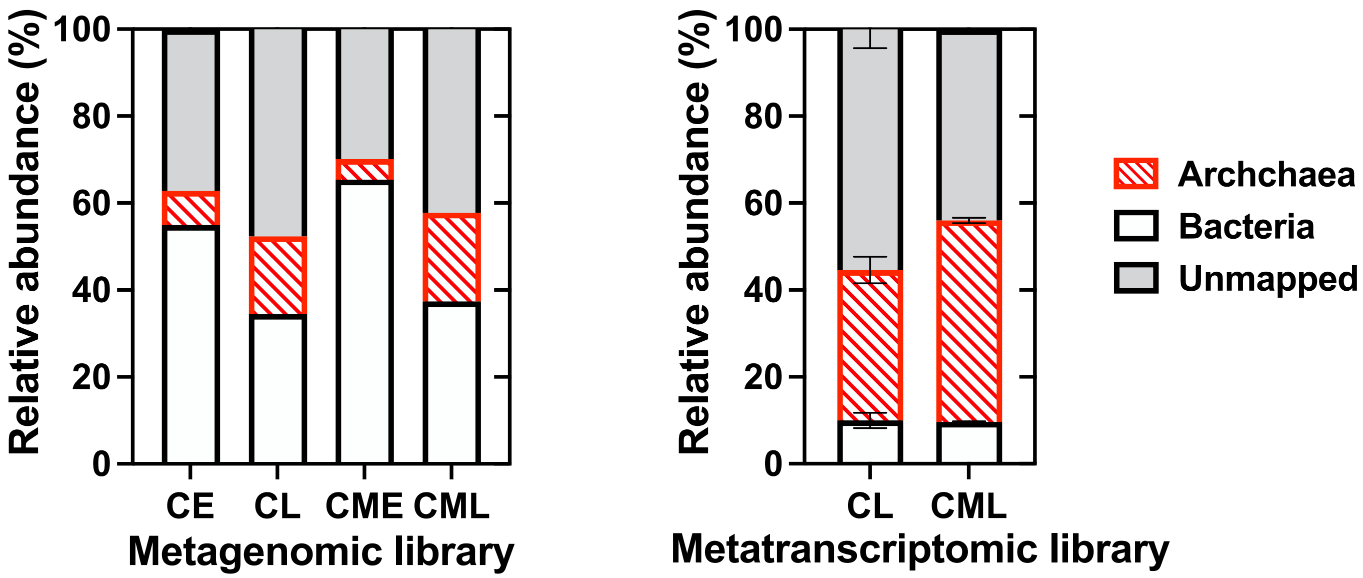

Figure S12. Relative abundance of bacteria and archaea in metagenomic and metatranscriptomic libraries at 5th transfer in the control group (C) and magnetite-amended group (CM) at stage E and stage L. Bar in figure B represents the means and standard deviations from three independent cultures.

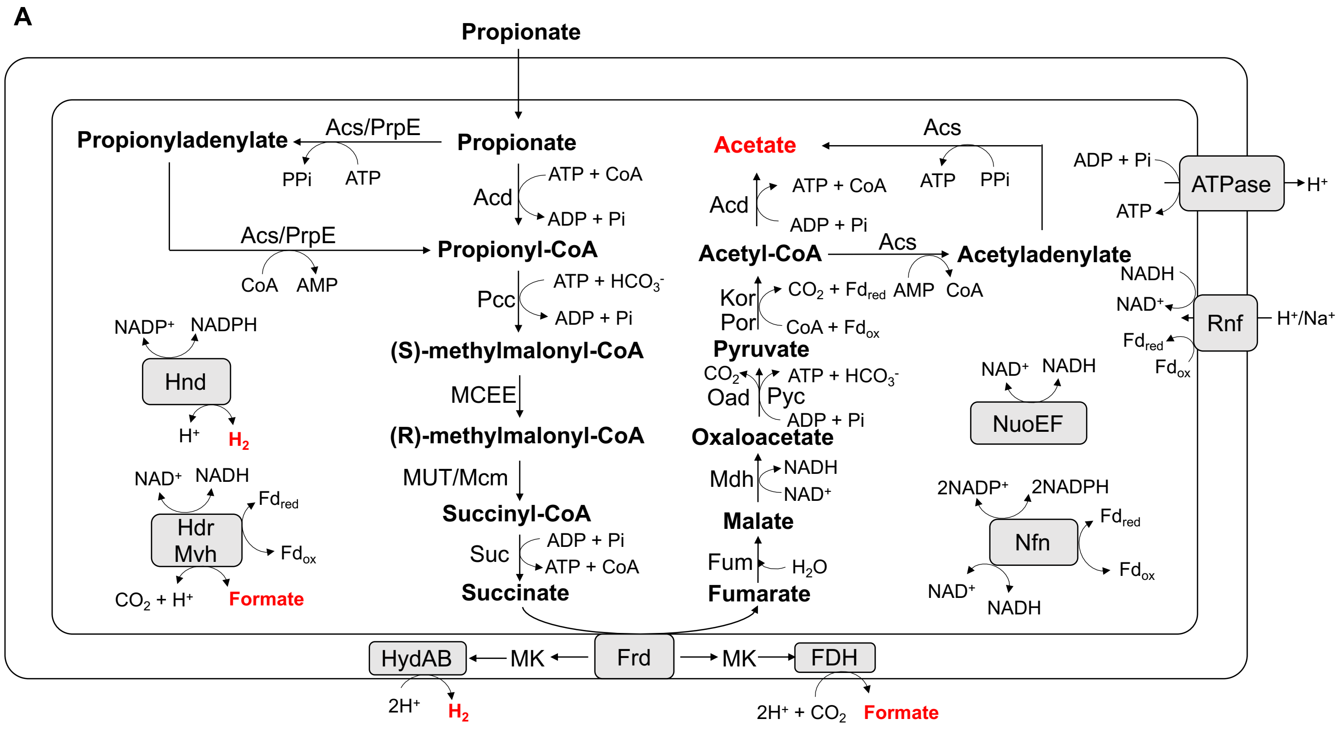

B

Log2TPM value: 0 — 5.5 — 11.0

Gene	Dea1 C	Dea1 CM	Dea2 C	Dea2 CM	Dea3 C	Dea3 CM
acs	3.62	-1.83	2.90	-0.25	-2.94	3.05
prpE	Missing		Missing		-2.40	4.67
acdAB	0.29	-2.11	-0.07	-0.66	-2.68	3.47
pcc	Missing		Missing		-3.51	2.91
mcee	Missing		7.63	4.47	1.16	9.34
mut	Missing		8.05	5.21	1.64	9.67
mcm	3.01	3.01	2.37	3.44	-1.72	2.09
suc	7.51	1.52	8.90	5.63	2.00	10.35
frd	8.41	3.03	7.36	5.30	2.34	10.02
fum	6.88	5.23	5.83	4.80	Missing	
mdh	8.14	1.32	7.21	2.42	1.86	7.97
pyc	0.90	-3.36	0.32	-1.60	-3.56	3.30
pycB	Missing		8.15	5.34	2.14	10.14
oadB	3.99	-2.59	2.75	-1.24	-5.93	-0.77
por	8.40	3.40	6.82	5.72	1.49	9.71
kor	2.97	-2.02	1.76	0.12	-0.86	4.73
hnd	4.47	-0.07	3.23	1.65	Missing	
hyd	6.20	1.27	5.28	2.68	Missing	
fdh	8.12	5.55	8.12	5.58	1.78	9.76
ATPase	7.52	2.78	8.51	4.12	3.11	9.03
nuoEF	4.88	-2.45	2.80	0.95	-3.38	2.70
rnf	6.03	3.62	6.08	3.58	0.98	9.02
nfn	7.75	2.04	6.76	4.20	1.41	9.02
hdr-mvh	2.46	0.28	1.95	-0.46	-1.02	5.86

Figure S13. Propionate oxidation pathway (A) and transcript levels of the core genes (B) in *Desulfobacterales* MAGs Dea1, Dea2, and Dea3 in mangrove microbial consortia in the presence or absence of magnetite. C: control group; CM: magnetite-amended group.

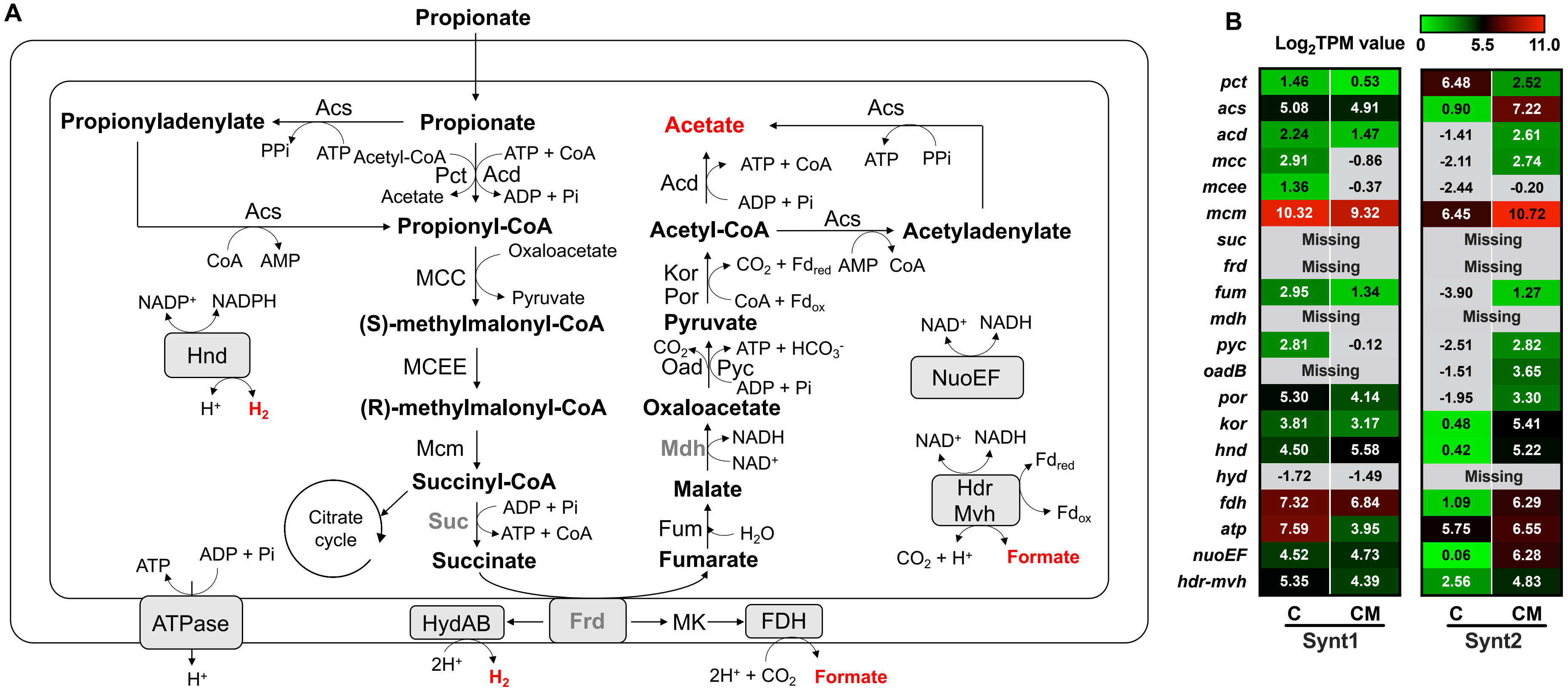

Figure S14. Propionate oxidation pathway (A) and transcript levels of the core genes (B) in *Smithellaceae* MAGs Synt1 and Synt2 in mangrove microbial consortia in the presence or absence of magnetite. C: control group; CM: magnetite-amended group.

Figure S15. Comparison of *Methanosarcina* sp. T3 transcriptome profiles in pure culture and in mangrove microbial consortia. (A) Multi-dimensional scaling (MDS) plot of *Methanosarcina* sp. T3 transcriptomes from pure cultures (P, PM) and mangrove microbial communities (C, CM) with or without magnetite addition. (B, C) Numbers of genes that were up-regulated (B) or down-regulated (C) in magnetite-amended groups compared with the control group, which were presented by Venn diagrams. PM, CM magnetite-amended group in pure culture and in mangrove microbial consortia; P, C non-magnetite control group in pure culture and in mangrove microbial consortia. Venn diagrams were produced by jvenn (Philippe Bardou, Jérôme Mariette, Frédéric Escudié, Christophe Djemiel and Christophe Klopp. jvenn: an interactive Venn diagram viewer. BMC Bioinformatics 2014, 15:293 doi:10.1186/1471-2105-15-293)

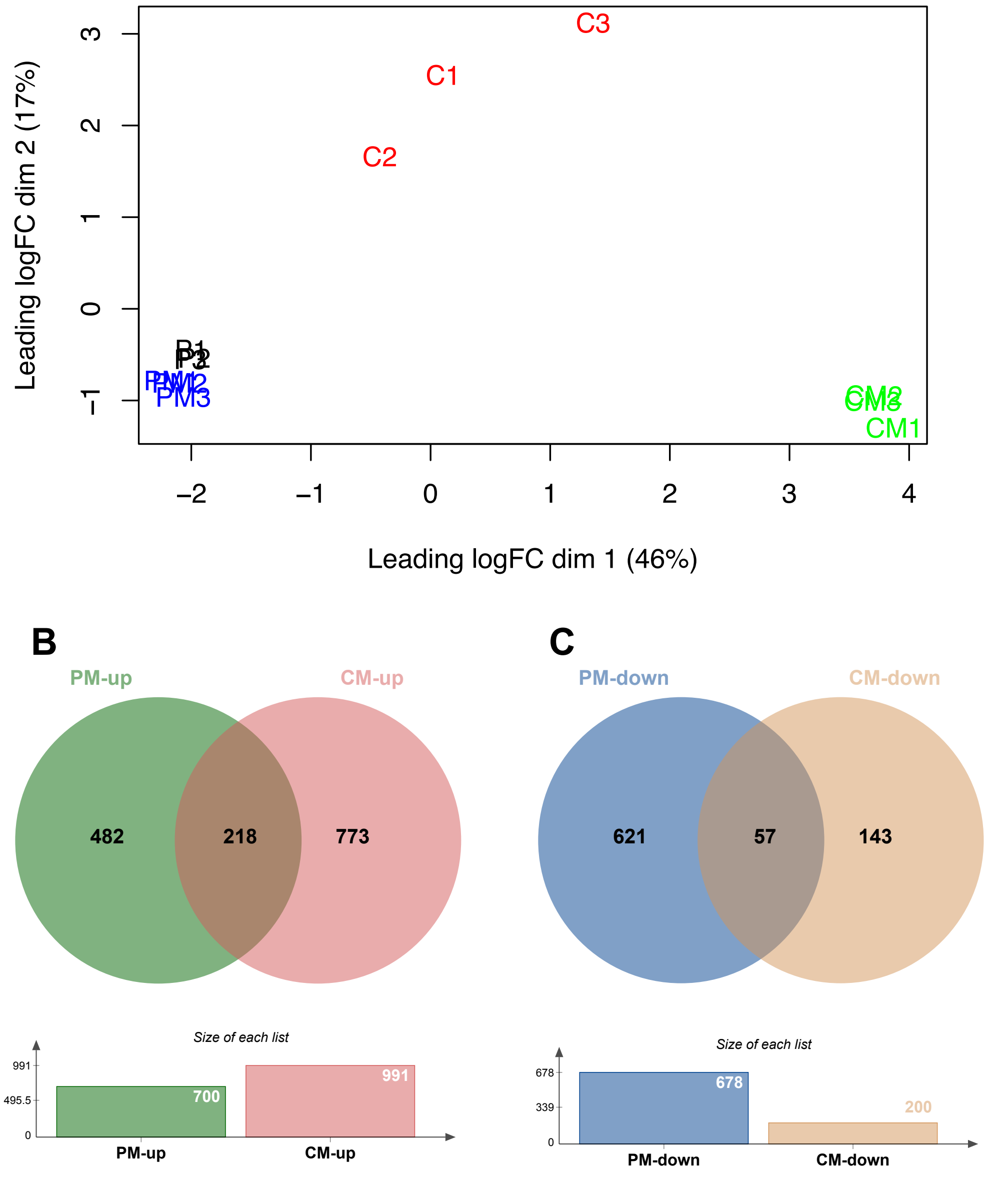

***Methanosarcina* sp. T3 (3 genes coding archaeal flagellin FlaB) proposed non-conductive**
>T3_3007 largest aromatics-free gap: 37 AAs; % aromatics: 11/182=6.4%
FTGLEAAIVLTAFVVVSAVFSYVILGAGFTTSDVTKATIDEGIKQTTSSVGLAGDVIAKSNNSKIDHVILTLQLTAGQSPINIGVDSVEGMM
VISYSDSAAYVANTTWTKEFVGNSDGDTILEQHEKVQITIEVPENSMLQSENMTDVVNREFRLEVKPNIGAILPVSRVTPPQIDAVMNLK

>T3_3026 largest aromatics-free gap: 48 AAs; % aromatics: 16/180=8.9%
FTGLEAAIVLIAFVVVAAVFSYVMLGAGFYTTQKSQEVVHTGVQQASSSLAPSGDVIVKGGASGVDEIIFYITSTAGGSAVDLDKTIVTYT
DVNDSITMEKDDGIWDYLASIDNGGANNLIESNEKYKITIDLTAATALNDQPVANEEIKIQVKPPEGAVLTLQRTMPASISSGVYYPVY

>T3_3027 largest aromatics-free gap: 76 AAs; % aromatics: 14/187=7.5%
FTGLEAAIVLVAFVVVAAVFSYVMLGAGFYTTQKSQEVVHTGVMQASSSVELSGDVIATGDAGASSTKLKNVTLCLQLTSGGSAVDMA
NTLIVVSAPGIAPTDLKLASDDVPTAALFGITGKYNANTNDLLERFEKFEVTVDLDAVGAAVDIEANDEFQLEIKPPQGASYTIHRQAPPSI
SAIMTLV

***Methanosarcina acetivorans* C2A (2 genes coding archaeal flagellin FlaB) proposed conductive**
>MA3062 largest aromatics-free gap: 26 AAs; % aromatics: 23/204=11.3%
FTGLEAAIVLIAFVVVAAVFSYVMLGAGFYTTQKSQEVVHTGVTQASSSIAPSGDVIVRGDAYGGNASQITFYVTNTAGGTSVDLDKTIVS
YTDDDDFVTQEYAVDTTATPVTKGPAAIPDDGAWGDTYGWVYNGTIVTNDDNLLEKGEKYKVVIDLDTFLGTGTLPTVNEKFKIEVKPP
EGAVLTISRTLPAALVTDNYYPVY

>MA3061 largest aromatics-free gap: 29 AAs; % aromatics: 17/179=9.5%
FTGLEAAIVLVAFVVVAAVFSYVMLGAGFYTTQKSQEVVHTGVQQASSSVELSGDVIARGNTTAANPADYSIDSITLCLQLTSGGSSVDM
DKTLIVYSDPDTAPTELTLDDGFTISTKYNANTDNLIDRAEKFDVSIDTSSYGIGPYNEFQLEIKPPQGASYTIHRTAPGSISAIMTLV

***Methanospirillum hungatei* JF-1 (1 genes coding archaeal flagellin FlaB) conductive**
>Mhun_3140 largest aromatics-free gap: 35 AAs; % aromatics: 18/164=11.0%
FSGLEAAIVLIAFVVVAAVFSYVMLGAGFFATQKSQEVTYSGMKQATSNLILDGMIYGSYSKGGSGLAQLYFYVKVPEGGETQDLKYVT
YLWTKENKAVTTLTSITPTNQQLNPGARVKVTITAPTGYKPIAGQKFVLEIKPKTGASTIVTRTLSDGYNGGVII

Figure S16. Amino acid arrangement of the mature archaeal flagellin FlaB in strain T3, typical type II *Methanosarcina M. acetivorans*, and *Methanospirillum hungatei* JF-1.

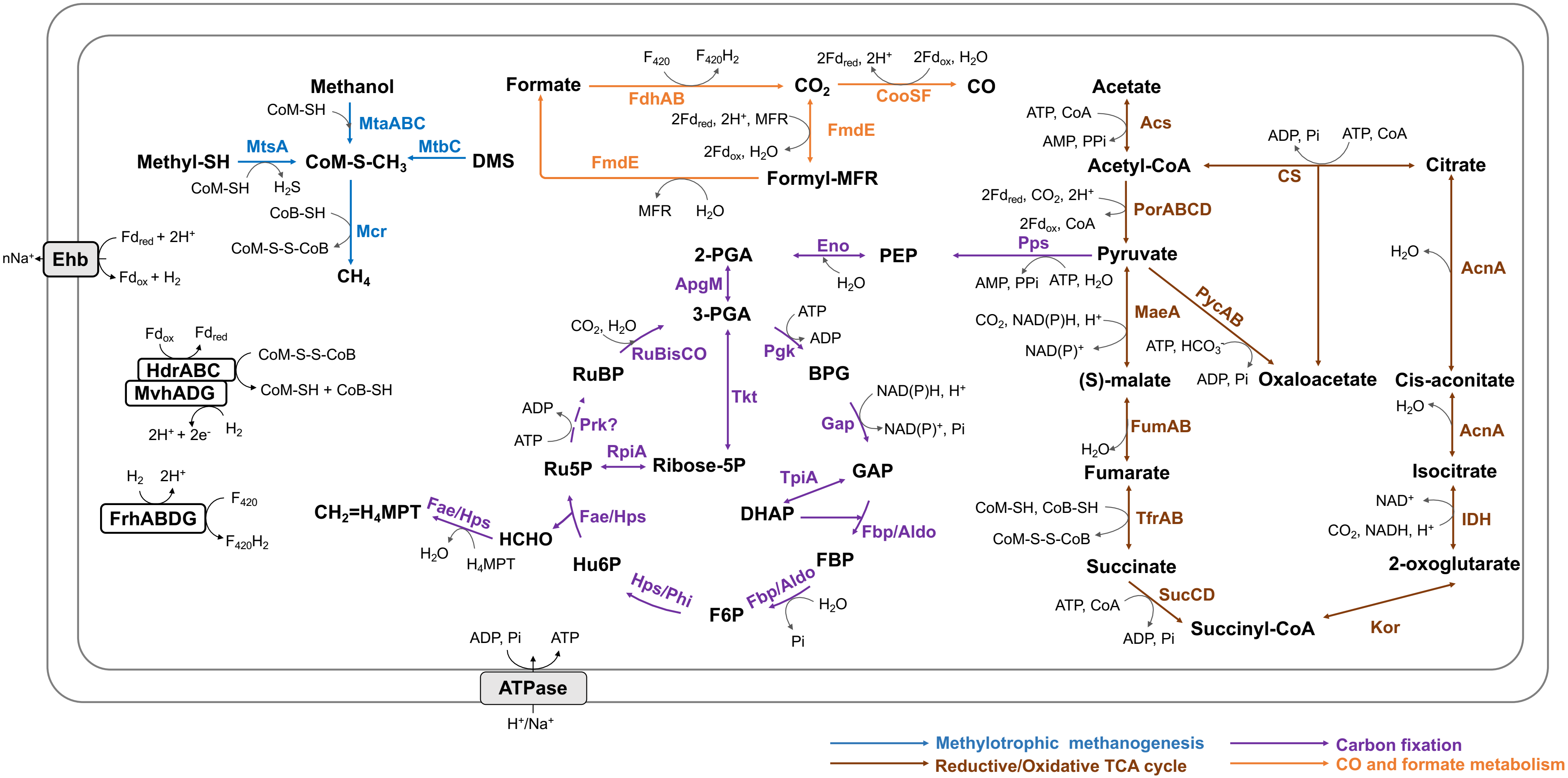

Figure S17. Proposed pathways of carbon metabolism and energy conservation in *Methanofastidiosales* Mef1 and Mef2 MAGs. CH3-S-CoM: 2-(methylthio) ethanesulfonate; DMS: dimethylsulfide; Methyl-SH: methanethiol; Formyl-MFR formylmethanofuran; PEP: phosphoenolpyruvate; 2-PGA: 2-phosphoglycerate; 3-PGA: 3-phosphoglycerate; BPG: 1,3-diphosphoglycerate; GAP: glyceraldehyde-3-phosphate; DHAP: dihydroxyacetone phosphate; FBP: fructose 1,6-bisphosphate; F6P: fructose 6-phosphate; Hu6P: D-arabino-3-hexulose-6-phosphate; Ru5P: ribulose-5-phosphate; HCHO: formaldehyde; RuBP: ribulose-1,5-bisphosphate; CH_2=H_4MPT: methylenetetrahydromethanopterin; Acetyl-CoA: acetyl-coenzyme A. Details regarding to each gene are provided in Table S8.
