# Supplementary material for: Magnetite drives microbial community restructuring and stimulates aceticlastic methanogenesis of type II Methanosarcina in mangrove sediments
Source: Microbiome. 2025 Jul 26;13:174. doi: 10.1186/s40168-025-02157-z (PMC12297640; doi:10.1186/s40168-025-02157-z)
Supplement: Supplementary file 5 — Supplementary Material 4. [file 40168_2025_2157_MOESM4_ESM.pdf]

Magnetite Drives Microbial Community Restructuring and Stimulates Aceticlastic Methanogenesis of Type II *Methanosarcina* in Mangrove Sediments

Jinjie Zhou[1], Cui-Jing Zhang[1], Dayu Zou[1], Chengxiang Gu[1], Meng Li[1*]

[1.] Archaeal Biology Centre, Synthetic Biology Research Center, Shenzhen Key Laboratory of Marine Microbiome Engineering, Key Laboratory of Marine Microbiome Engineering of Guangdong Higher Education Institutes, Institute for Advanced Study, Shenzhen University, Shenzhen 518060, China

[*] Correspondence: Meng Li, E-mail: limeng848@szu.edu.cn

Bacteria responsible for lactate degradation to propionate and acetate

Clostridium Clos MAG was the most abundant bacterial MAG under both conditions, accounting for 12.1% and 11.1% of the metagenomic libraries at stage E in the control and magnetite-amended cultures, respectively (Figure 2). The genus *Clostridium*, within the phylum *Bacillota*_A, are well-characterized lactate-utilizing bacteria that produce acetate, CO_2, and hydrogen as fermentation end products, and species are also capable of converting lactate to propionate and/or butyrate [1, 2]. The Clos MAG encoded a complete acrylate pathway for propionate production from lactate, as well as lactate dehydrogenase (LDH) for converting lactate to pyruvate and ultimately to acetate (Table S5). Both 16S rRNA gene amplicon and metagenomic analyses consistently highlighted the critical role of *Clostridium* in lactate degradation at the early cultivation stage, and magnetite addition did not appear to affect its metabolic activity.

Nonetheless, magnetite addition altered the relative abundance of several bacterial MAGs in the metagenomic dataset (Figure 2). Two MAGs, Cor1 and Cor2, were exclusively enriched in the magnetite-amended group, especially at stage E. These MAGs were assigned to the phylum *Actinomycetota*, class *Coriobacteriia*, order *Anaerosomatales*, and family *Anaerosomataceae*. Both MAGs encoded putative LDH (COG0039) and genes essential for pyruvate metabolism but lacked the core genes to ferment lactate to propionate (Table S5). The 16S rRNA gene (1,512 bp) retrieved from Cor1 showed 95.88% and 94.22% identity with *Parvivirga hydrogeniphila* Es71-Z0120^T and *Anaerosoma tenue* M08DHB^T, respectively. These two reference strains

are chemolithotrophic Fe(III) reducers capable of using hydrogen or formate as electron donors [3]. We speculate that Fe(III) in magnetite may serve as a terminal electron acceptor, supporting the growth of these microorganisms in our system.

Another MAG, Dem3 (affiliated with the phylum *Desulfobacterota*, genus *Trichloromonas*), was also selectively enriched in the magnetite-amended cultures, accounting for 6.5% and 4.2% of metagenomic reads at stages E and L, respectively, but less than 0.08% in the control group. *Trichloromonas* has recently been identified as a dominant electroactive microorganism in anode biofilms, playing a key role in extracellular electron transfer and current generation [4], and mineral amendment (hematite) can further increase its relative abundance [5]. Thus, the consistent enrichment of *Trichloromonas* in the magnetite-amended cultures implies that this genus may participate in extracellular electron transfer (EET), potentially coupling the oxidation of organic compounds to Fe(III) reduction in magnetite, using lactate as the electron donor at stage E.

By contrast, Cloa3 (100% completeness, 1.1% contamination) was only enriched in the control group, accounting for 6.9% and 10.6% in the metagenomic library at stage E and L, whereas less than 0.5% in the magnetite-amended group. This MAG was assigned to the phylum *Cloacimonadota*, class *Cloacimonadia*, order *Cloacimonadales*, family *Cloacimonadaceae*, and genus UBA5456 according to GTDB-Tk database. The phylum *Cloacimonadota* represents an uncultured bacterial lineage frequently detected in anaerobic wastewater and landfill systems [6]. which is predicted with acetogenic and mixed fermentative flavin-bifurcation-based anaerobic respiratory lifestyle,

forming electron transport chain with Rnf complex and ATP synthase for energy conservation [7], and interaction with hydrogen-consuming partners for example hydrogenotrophic methanogen [8]. In our study, the Cloa3 MAG encoded a putative malate/L-lactate dehydrogenase (COG2055, Cloa3_1772) and a complete pyruvate oxidation pathway, but no enzymes associated with lactate-to-propionate conversion were detected. Thus, this MAG is likely involved in converting lactate into acetate and hydrogen in the control group. Genomic analysis revealed the presence of three types of cytoplasmic [FeFe] hydrogenases (groups A1, C1, and C3), predicted to catalyze hydrogen production using oxidized ferredoxin (A1) or PAS domains (C1, C3) as electron acceptors. The genome also encoded a complete Rnf complex (*rnfBAEGDC*, Cloa3_465-470) and a nearly complete Nqr complex (*nqrBCDEF*, Cloa3_1558-1562, lacking NqrA) to perform reverse electron transport, regenerate reduced ferredoxin and NADH, and maintain a transmembrane Na^+/H^+ gradient for energy conservation. A previous study reported that magnetite addition significantly decreased the transcriptional activity of *Cloacimonadota* in a propionate-fed anaerobic reactor [9]. Together with our findings, this suggests that magnetite may negatively affect the growth and lactate metabolism of *Cloacimonadota*, although further investigation is still needed.

Possible routes for *Candidatus* Methanofastidiosales to acquire carbon

Candidatus Methanofastidiosales is an uncultivated archaeal lineage frequently detected in various methanogenic environments including the mangrove sediments that

used as inoculum in our study [10]. Previous studies indicate that this lineage lacks genes for Wood-Ljungdahl pathway but rely on methyltransferases for methane production using methyl compounds as substrates [11, 12]. In this study, the absence of methyl compounds in the cultivation media raises the question of how this archaeal lineage survived and conserved energy in the enriched cultures even after five serial transfers (Figure 2), even though their transcript activity is low (Figure 3).

Two MAGs Mef1 and Mef2 (88.85-89.45% completeness, and 2.16-2.65% contamination) were recovered from the enrichments, both assigned to the order *Methanofastidiosales* and the family NM3 based on GTDB taxonomy. Considering the low transcriptomic activities of this lineage in both control and magnetite-amended enrichments (Figure 3), we primarily focused on metagenomic data, reconstructing the carbon metabolism and energy conservation pathways based on two *Methanofastidiosales* MAGs (Figure S17, Table S8).

Similar to previous study [11], both *Methanofastidiosales* MAGs lack core genes for Wood-Ljungdahl pathway, such as *fwd*, *ftr*, *mch*, *mtd, mer*, or *mtr*. Instead, genes encoding methanol: CoM methyltransferase MtaABC, methylthiol: CoM methyltransferase MtsA, and dimethylamine corrinoid protein MtbC were identified, indicating their potential for methylotrophic methanogenesis using methanol, methylated sulfides, and dimethyl sulfide as substrates. Two cytoplasmic hydrogenases, MvhADG and FrhADBG were identified in both MAGs. Using H_2 as the electron donor, MvhADG works with hederdisulfide reductase, forming HdrABC/MvhADG complex to reduce heterodisulfide and ferredoxin, while FrhADBG is responsible for coenzyme

F_{420} reduction. Genes encoding the membrane-bound energy-conserving hydrogenase Ehb were identified, which is predicted to generate sodium gradient for energy conservation. In addition, both *Methanofastidiosales* MAGs have genes encoding the RuBisCO-mediated reductive hexulose phosphate (RHP) pathway, with the exception of phosphoribulokinase (Prk, EC 2.7.1.19). While a gene encoding phosphoribulokinase/uridine kinase family (PF00485, PRK) was identified in the Mef2 MAG, which may function as Prk to convert ribulose-5-phosphate (Ru5P) to ribulose-1,5-bisphosphate (RuBP). RuBisCO-mediated RHP pathway is widely distributed in various methanogens and is likely associated with methanogenesis and/or Wood-Ljungdahl pathway [13]. However, formaldehyde-derived methylene-H_4MPT could not further enter the downstream pathways, as they lack core genes. Therefore, we are unable to link CO_2 fixation with CH_4 production for energy conservation. Another hypothesis is that the growth and energy conservation of *Methanofastidiosales* are not coupled with methane production, although further verification remains to be defined. Nevertheless, we hypothesize that *Methanofastidiosales* utilized CO_2 and/or acetate as carbon sources via the RuBisCO-mediated RHP and/or oxidative/reductive TCA pathways. Although the enzyme activity was not verified, it is suspected that *Methanofastidiosales* acquired carbon via the RuBisCO-mediated RHP pathway and/or the reductive/oxidative TCA cycle, using CO_2 and/or acetate as carbon source instead of methyl compounds.

References

1. Tao Y, Hu X, Zhu X, Jin H, Xu Z, Tang Q, et al. Production of Butyrate from Lactate by a Newly Isolated *Clostridium* sp. BPY5. Appl Biochem Biotechnol. 2016;179(3):361-74.
2. Reichardt N, Duncan SH, Young P, Belenguer A, McWilliam Leitch C, Scott KP, et al. Phylogenetic distribution of three pathways for propionate production within the human gut microbiota. ISME J. 2014;8(6):1323-35.
3. Khomyakova MA, Zavarzina DG, Merkel AY, Klyukina AA, Pikhtereva VA, Gavrilov SN, et al. The first cultivated representatives of the actinobacterial lineage OPB41 isolated from subsurface environments constitute a novel order *Anaerosomatales*. Front Microbiol. 2022;13:1047580.
4. Wang N, Yang Y, Xu K, Long X, Zhang Y, Liu H, et al. Distinguishing anaerobic digestion from electrochemical anaerobic digestion: Metabolic pathways and the role of the microbial community. Chemosphere. 2023;326:138492.
5. Jiang X, Gao X, Yang K, Hu J, Cao X, Sakamaki T, et al. Promotion and mechanism of defective hematite on the power generation and phenanthrene degradation of soil microbial fuel cells. Environmental Engineering Research. 2024;30(1):240135-0.
6. Dyksma S, Gallert C. *Candidatus* Syntrophosphaera thermopropionivorans: a novel player in syntrophic propionate oxidation during anaerobic digestion. Environ Microbiol Rep. 2019;11(4):558-70.
7. Johnson LA, Hug LA. Cloacimonadota metabolisms include adaptations in engineered environments that are reflected in the evolutionary history of the phylum. Environ Microbiol Rep. 2022;14(4):520-9.
8. Williams TJ, Allen MA, Berengut JF, Cavicchioli R. Shedding Light on Microbial "Dark Matter": Insights Into Novel Cloacimonadota and Omnitrophota From an Antarctic Lake. Front Microbiol. 2021;12:741077.
9. Dyksma S, Gallert C. Effect of magnetite addition on transcriptional profiles of syntrophic Bacteria and Archaea during anaerobic digestion of propionate in wastewater sludge. Environ Microbiol Rep. 2022;14(4):664-78.
10. Zhang CJ, Pan J, Liu Y, Duan CH, Li M. Genomic and transcriptomic insights into methanogenesis potential of novel methanogens from mangrove sediments. Microbiome. 2020;8(1):94.
11. Nobu MK, Narihiro T, Kuroda K, Mei R, Liu WT. Chasing the elusive Euryarchaeota class WSA2: genomes reveal a uniquely fastidious methyl-reducing methanogen. ISME J. 2016;10(10):2478-87.
12. Borrel G, Adam PS, McKay LJ, Chen LX, Sierra-Garcia IN, Sieber CMK, et al. Wide diversity of methane and short-chain alkane metabolisms in uncultured archaea. Nat Microbiol. 2019;4(4):603-13.
13. Kono T, Mehrotra S, Endo C, Kizu N, Matusda M, Kimura H, et al. A RuBisCO-mediated carbon metabolic pathway in methanogenic archaea. Nat Commun. 2017;8:14007.
